# Effect of identified non-synonymous mutations in *DPP4* receptor binding residues among highly exposed human population in Morocco to MERS-CoV through computational approach

**DOI:** 10.1371/journal.pone.0258750

**Published:** 2021-10-14

**Authors:** Anass Abbad, Latifa Anga, Abdellah Faouzi, Nadia Iounes, Jalal Nourlil

**Affiliations:** 1 Medical Virology and BSL-3+ Laboratory, Institut Pasteur Morocco, Casablanca, Morocco; 2 Laboratoire d’Ecologie et d’Environnement, Faculté des Sciences Ben M’sik, Université Hassan II – Casablanca, Casablanca, Morocco; United Arab Emirates University, UNITED ARAB EMIRATES

## Abstract

Dipeptidyl peptidase 4 (*DPP4*) has been identified as the main receptor of MERS-CoV facilitating its cellular entry and enhancing its viral replication upon the emergence of this novel coronavirus. *DPP4* receptor is highly conserved among many species, but the genetic variability among direct binding residues to MERS-CoV restrained its cellular tropism to humans, camels and bats. The occurrence of natural polymorphisms in human *DPP4* binding residues is not well characterized. Therefore, we aimed to assess the presence of potential mutations in *DPP4* receptor binding domain (RBD) among a population highly exposed to MERS-CoV in Morocco and predict their effect on *DPP4* –MERS-CoV binding affinity through a computational approach. *DPP4* synonymous and non-synonymous mutations were identified by sanger sequencing, and their effect were modelled by mutation prediction tools, docking and molecular dynamics (MD) simulation to evaluate structural changes in human *DPP4* protein bound to MERS-CoV S1 RBD protein. We identified eight mutations, two synonymous mutations (A291 =, R317 =) and six non-synonymous mutations (N229I, K267E, K267N, T288P, L294V, I295L). Through docking and MD simulation techniques, the chimeric *DPP4* –MERS-CoV S1 RBD protein complex models carrying one of the identified non-synonymous mutations sustained a stable binding affinity for the complex that might lead to a robust cellular attachment of MERS-CoV except for the *DPP4* N229I mutation. The latter is notable for a loss of binding affinity of *DPP4* with MERS-CoV S1 RBD that might affect negatively on cellular entry of the virus. It is important to confirm our molecular modelling prediction with *in-vitro* studies to acquire a broader overview of the effect of these identified mutations.

## Introduction

The Middle East Respiratory Syndrome of Coronavirus (MERS-CoV) is a zoonotic enveloped single-strand positive RNA virus. This novel emerging betacoronavirus was isolated for the first time in 2012 in a human patient with a severe pneumonia [1]. MERS-CoV is now of global public health concern, responsible for over 2581 cases with a high fatality rate of 34.4%, as of the end of January 2021 [2]. Dromedary camels have been identified as the zoonotic source for human MERS-CoV infection following close contact with these animals [3, 4]. Sustained human-to-human transmission has been so far limited to health settings [5, 6]. Sporadic cases of MERS-CoV disease have so far been restricted to the Arabian peninsula [7]. However, MERS-CoV does appear to transmit asymptomatically in North and Sub-Saharan Africa as detected by a seroprevalence of neutralizing antibodies of 0.18% in comparison to the Arabian peninsula (0.72%) [8]. Since Africa has by far the largest numbers of dromedary camels, the lack of zoonotic disease is surprising [9].

MERS-CoV exploits dipeptidyl peptidase 4 (*DPP4*, also known as *CD26*) for cellular entry and viral replication [10]. *DPP4* forms a homodimer. Each subunit contains two domains: α/β-hydrolase domain and a β-propeller domain. The full-length *DPP4* is a type II transmembrane protein in which amino acids 7–28 constitute the membrane-spanning region. The α/β-hydrolase domain, located closest to the membrane, consists of amino acids 39–51 and 506–766, and contains the active triad Ser630, Asp708 and His740 [11]. Residues 55–497 form the eight-bladed β-propeller domain, and it has a glycosylation-rich region comprising blades II—V while blades VI—VIII are in a cysteine-rich region. Each blade shows a 4-stranded antiparallel β sheet motif, and blade IV has an additional antiparallel sheet (Asp230-Asn263) between strands 3 and 4 of blade IV [11, 12]. According to structural analyses, the MERS-CoV spike protein’s receptor binding domain (RBD) mediates viral infections by binding restrictively to blades IV and V of the N-terminal β-propeller domain of the *DPP4* receptor [12]. The resolution of full protein crystallographic structure of the *DPP4* binding to MERS-CoV S protein complex mediated the identification of 16 amino acids residues in *DPP4* receptor binding domain (RBD) interacting directly with MERS-CoV S protein [13, 14]. Humans, camels and bats use the *DPP4* receptor for binding with MERS-CoV S protein [15]. The genetic variability of these *DPP4* amino acids residues in direct contact with MERS-CoV among animal species was a determinant factor for cellular non-permissiveness of MERS-CoV among some animal species [16].

The *DPP4* cell surface receptor is widely expressed in human tissues. It is involved in diverse cellular functions, playing a critical role in physiologic glucose homeostasis [17]. Its enzymatic activity has been implicated in the regulation of the biologic activity of multiple hormones, chemokines and T-cell activity [18–20]. Serious human health conditions such as diabetes and myocardial infarction have been strongly associated with the presence of genomic mutations or SNPs in the *DPP4* gene [21, 22]. However, there is an urgent need to address the lack of information on genomic human variation of *DPP4*, specifically on the binding area to MERS-CoV S protein that might affect the *DPP4* –MERS-CoV S protein complex binding affinity by inducing structural conformation changes.

Therefore, throughout this study, we aimed to identify a potential presence of mutations in *DPP4* receptor binding domain among a population in Morocco highly exposed to dromedary camels and thus to MERS-CoV and predict their effect on *DPP4* –MERS-CoV binding affinity through an *in-silico* approach.

## Materials and methods

### Study population and field sampling

The genomic characterization of *DPP4* RBD was conducted on human subjects (n = 100) belonging to a human population with exposure to dromedary camels, and thus to MERS-CoV, in southern regions of Morocco. This population had a seroprevalence of MERS-CoV neutralising antibodies of 0.83% [8]. These selected human subjects belonged to three exposure risk categories: general population without direct exposure to camels (n = 34), camel herders (n = 33) and slaughterhouse workers (n = 33) having a direct exposure to camels. Each of the selected participants provided a whole blood sample collected in 5 ml EDTA tubes with a signed informed consent to participate in MERS-CoV related studies (IRB reference number 55/16).

### Molecular analysis

#### DNA extraction and DNA quality and quantification

Human genomic DNA was isolated from 200 μl of whole blood samples using TRIzol LS reagent (Invitrogen, Thermo Fischer scientific) according to the manufacturer’s instructions. Following DNA isolation, the DNA concentration of each sample was measured using NanoDrop 2000/2000c spectrophotometer (Invitrogen, Thermo Fischer scientific).

#### Primers design

The *DPP4* RBD is coded by four exons of *DPP4* human gene: exon 9, exon 10, exon 11 and exon 12. Thus, we designed four sets of intronic primers for conventional PCR and sequencing to amplify targeted exonic regions, using Primer Express 3.0.1 (Applied Biosystem, Foster City, CA, USA) and the reference sequence of the *hDPP4* gene (NC_000002.12) retrieved from GenBank database. Designed primers were evaluated for specificity using MFEprimer-3.0 online tool [23], and are detailed in Table 1.

**Table 1:** Summary table of designed primers for amplification and sequencing of *hDPP4* gene exons 9, 10, 11 and 12.

Primer ID	Nucleotide primer sequence (5’ → 3’)	Amplicon size (bp)
***DPP4*-Exon 9- Fw**	TGCCAAAGCCAATTTATCAGCT	356 bp
***DPP4*-Exon 9- Rv**	TGCCAGATGCTGTTGACTTCA	
***DPP4*-Exon 10- Fw**	GGTTGCATTTCATGACTCTCCC	388 bp
***DPP4*-Exon 10- Rv**	GGGAGGCTGTGATCCACTTT	
***DPP4*-Exon 11- Fw**	CCAAGGTCTGGCAATAGTCA	382 bp
***DPP4*-Exon 11- Rv**	CCTCGGGATGGCAGGTTATC	
***DPP4*-Exon 12- Fw**	GAGCTTCCAGAAGGACCCAG	352 bp
***DPP4*-Exon 12- Rv**	ACGTATCACTTAGAGCCCTAGT	

#### Conventional PCR

The amplification of the targeted *hDPP4* exons of each DNA sample was performed separately in a final volume of 25 μL containing the 1X Master Mix (SuperMix, Invitrogen, Thermo Fischer scientific), 0.4 μM of each primer and 250–300 ng of DNA. In each serial, a no template control (NTC) without DNA or RNA was included. PCR amplification was performed using a GeneAmp PCR system 2720 (Applied Biosystem, Foster City, CA, USA), under the following PCR cycling conditions: 1 cycle of 94°C for 4 minutes; 40 cycles of 94°C for 30 secs, 50°C for 30 secs, 72°C for 30 secs and 1 cycle of 72°C for 5 minutes. Amplification products were analyzed by electrophoresis method on a 2% Agarose gel and visualization using a molecular imager (Gel Doc XR with the Quantity-One software, BioRad).

#### Sanger sequencing and data analysis

The amplicons of each target were purified using PureLink PCR Purification kit (Invitrogen, Thermo Fischer scientific) according to the manufacturer’s instructions. Sequencing PCR reaction was performed using BigDye Terminator kit v. 3.1 (Applied Biosystem, Foster City, CA, USA) and purified with ethanol/EDTA precipitation as described by the manufacturer. The purified products of the cycle sequencing were analysed on the capillary electrophoresis ABI 3130xl Genetic Analyser (Applied Biosystem, Foster City, CA, USA). This process was carried out at the *UATRS Genomic Centre*, *CNRST*, *Rabat*, *Morocco*.

The retrieved sequences were analysed for nucleotide mutations associated with amino acid binding residues of the *hDPP4* receptor with MERS-CoV S protein using BioEdit v.7.1.9 software [24]. Each sequence was aligned with the reference *hDPP4* gene (NC_000002.12) using ClustalW algorithm.

### Statistical analysis

The statistical significance of the presence of a mutation in the interacting amino acid residues of *DPP4* receptor by sex, exposure group, and MERS-CoV serological profile was analyzed using the Fisher test, Risk Ratio was determined. Statistical significance was defined as p <0.05.

### Molecular modelling analysis

To evaluate the effect of non-synonymous nucleotide mutation identified on amino acid residues participating directly in *DPP4* binding with MERS-CoV, we followed two molecular modelling approaches. The first *in-silico* approach was based on mutation effect prediction tools Mutabind2 [25] and DynaMut [26]. Then a second approach based on molecular modelling of each identified mutation on conformation stability and binding affinity of the complex through docking and molecular dynamics. Thus, we selected the EM-cryo structure of *DPP4* –MERS-CoV S1 RBD protein complex (PDB: 4L72) from PDB public database. Crystal water molecules were removed from the PDB file using PyMOL [27] before downstream application.

#### Prediction tools for non-synonymous mutation effect

We predicted first the effect of non-synonymous mutations by evaluating the binding affinity of *DPP4* mutants to the MERS-CoV S1 RBD in comparison with wild type complex using Mutabind2 and DynaMut webservers using 4L72 PDB file. These computational tools compare binding affinities after mutations to predict whether they stabilize or destabilize the protein–protein interaction by determining the overall change in binding free energies (ΔΔG). The effect of non-synonymous mutations in protein—protein complex stability and estimation of change in the folding free energy ($\Delta \Delta G^{Destabilizing}=\;\Delta G_{mutated}^{Destabilizing}-\;\Delta G_{wild}^{Destabilizing}$) was predicted using DynaMut webserver. A positive and negative outcome correspond to stabilizing and destabilizing mutations predicted to decrease and increase folding free energy respectively. Whereas, changes in binding affinity ($\Delta \Delta G^{Binding}=\;\Delta G_{mutated}^{Binding}-\;\Delta G_{wild}^{Binding}$) upon single mutation was predicted with Mutabind2 online tool, as a positive and negative outcome correspond to destabilizing and stabilizing mutations predicted to decrease and increase binding affinity correspondingly for DynaMut and Mutabind2 respectively.

#### Structure preparation for molecular dynamics approach

A mutant model of the *DPP4* carrying one of each non-synonymous mutations was created using the Mutagenesis tool in PyMOL software [27]. The models were named as the following respectively to wild type or the name of present mutation in *DPP4* protein; 4L72-WT, 4L72-N229I, 4L72-K267E, 4L72-K267N, 4L72-T288P, 4L72-L294V, 4L72-I295L. Afterward, the wild and mutant *DPP4* models were validated using an online MolProbity server for Ramachandran plot analysis [28], Verify 3D [29], and ProSA analysis [30].

#### Molecular docking of MERS-CoV S1 RBD with wild and mutant models of *DPP4*

Protein docking was implemented through the online docking webserver HADDOCK 2.4 (https://wenmr.science.uu.nl/haddock2.4/) [31]. For the docking of MERS-CoV S1 RBD (PDB: 4L72_B) to *DPP4* (4L72_A), we submitted the corresponding monomeric crystal structure prepared models to HADDOCK 2.4 webserver to obtain the complex model. We defined active amino acid residues positions directly involved in the interaction based on the *DPP4* –MERS-CoV S1 RBD interaction interface of the crystal structure complex. Sixteen residues for *DPP4* protein and eighteen residues for MERS-CoV S1 RBD were selected as active residues for complex docking [13, 14]. Passive residues for both proteins of the complex were defined by default parameters within a radius of 4 Å. We selected the top of the ten generated *DPP4* –MERS-CoV S1 RBD complex with the lowest *z-score* and *HADDOCK score* for molecular dynamics analysis.

#### Molecular dynamic (MD) simulation

A MD simulation was performed for all the *DPP4* –MERS-CoV S1 RBD wild and mutant models using Gromacs 5.1.3 program [32, 33] with CHARMM36 force field [34]. Each system was first solvated in a dodecahedron box of SPC water molecules with a box-system minimal distance of 1.0 nm between the solute and the wall of the box. The system was neutralized with an appropriate amount of sodium ions by replacing water molecules. The minimization was carried on the system through 5000 steps of the steepest descent. The systems were then equilibrated (500 ps for NVT heating to 300 K, followed by 500 ps for NPT), applying the position restraints on protein complex with periodic boundary conditions. The pressure and temperature were set at 1 bar and 300 K pressure using Parrinello-Rahman and Berendsen coupling methods, respectively. Particle Mesh Ewald (PME) computed the long-range electrostatic interaction with a distance of 1.2 nm for short-range non-bonded cut-off. All covalent bonds including heavy atom-H were constrained by the LINCS algorithm. Finally, the system was further equilibrated to carry out 150 ns MD simulations at a constant temperature of 300 K, maintained by the v-rescale thermostat and a time step of 2 fs.

The root-mean square deviation (RMSD), root-mean-square fluctuation (RMSF), radius of gyration (Rg), solvent accessible surface area (SASA),hydrogen bonds (H-Bonds) and principal component analysis (PCA) were analyzed throughout the trajectory using the *gmx rmsd*, *gmx rmsf*, *gmx gyrate*, *gmx sasa*, *gmx hbond*, *gmx covar and gmx anaeig* respectively, built-in function of the GROMACS software. The graphs were plotted using Xmgrace tool.

## Results

### Population study demographics

The selected population is characterized by a male/female sex-ratio of 3:1, with camels herders (n = 34), slaughterhouse workers (n = 31) and the general population (n = 35) having a sex-ratio of 3:0, 15:0 and 1:1 respectively. This population is characterized by an average age of 40 years [16–76 years] and a median age of 38 years. The most representative age groups in this study were 21–30 years (23%) and 41–50 years (22%). While the age groups ≤ 20 years and ≥ 70 years were the least represented in this population with 9% and 3% respectively (Table 2).

**Table 2:** Middle East respiratory syndrome coronavirus (MERS-CoV) study population demographics, dromedary exposure and serological profile by study group.

Characteristics	Camels herders (n = 34)	Slaughterhouse workers (n = 31)	General population (n = 35)	Total (n = 100)
*Sex*				
Male	34	29	13	76
Female	0	02	22	24
*Age*				
≤ 20 years	01	03	03	9
20 < years ≤ 30	06	10	08	24
31 < years ≤ 40	08	05	07	20
41 < years ≤ 50	08	06	08	22
51 < years ≤ 60	06	03	03	12
61 < years ≤ 70	03	01	06	10
> 70 years	02	01	00	03
*Serological MERS-CoV profile using ELISA assay* ^a^ [8]				
Negative	18	21	16	55
Borderline positive	11	06	06	23
Positive	05	08	09	22
*Serological MERS-CoV profile using neutralisation assays (ppNT + PRNT)* ^b^ [8]				
Negative	34	34	28	96
Positive	00	03	01	04

^a^ Optical Density (OD) ratios recommended as cut off for positive (1.1) and borderline (0.8) by the ELISA kit manufacturer.

^b^ Positives defined as ppNT-positive and confirmed by ≥ 90% reduction of plaque counts in PRNT test.

This population study has a MERS-CoV seroprevalence by ELISA technique of 22%, while 23% were borderline positive and 55% were negative. However, neutralizing antibodies in this population have established a MERS-CoV seroprevalence of 4% (Table 2) [8].

### Genetic characterization of *DPP4* gene

The molecular characterization of the exons of interest (exon 9–12) through Sanger sequencing permitted the identification of six non-synonymous mutations, inferring a change in amino acid residues of the *DPP4* protein chain structure. These mutations are mainly identified on *DPP4* exon 9; c.686C> T (p. Asn229> Ile, N229I) and exon 10; c.801G> C (p. Lys267Asn, K267N), c.799A> G (p. Lys267Gln, K267E), c.862A> C (p. Thr288Pro, T288P), c.880T> G (p. Leu294Val, L294V) and c.884T> A (p. Ile295Leu, I295L). Moreover, two synonymous mutations were also identified on exon 10 and exon 11, and described respectively as follows: c.872T> C (p.Ala291 =, A291 =) and c.951G> A (p.Arg317 =, R317 =). The synonymous mutation R317 = was the most prevalent (3%) among the population studied, while the non-synonymous mutation L294V was identified in 2% of the population. The other described mutations respectively accounted for 1% of the study population. However, no participant subject in this study carried more than one mutation at the interaction residues level of the *hDPP4* gene.

**Fig 1:**
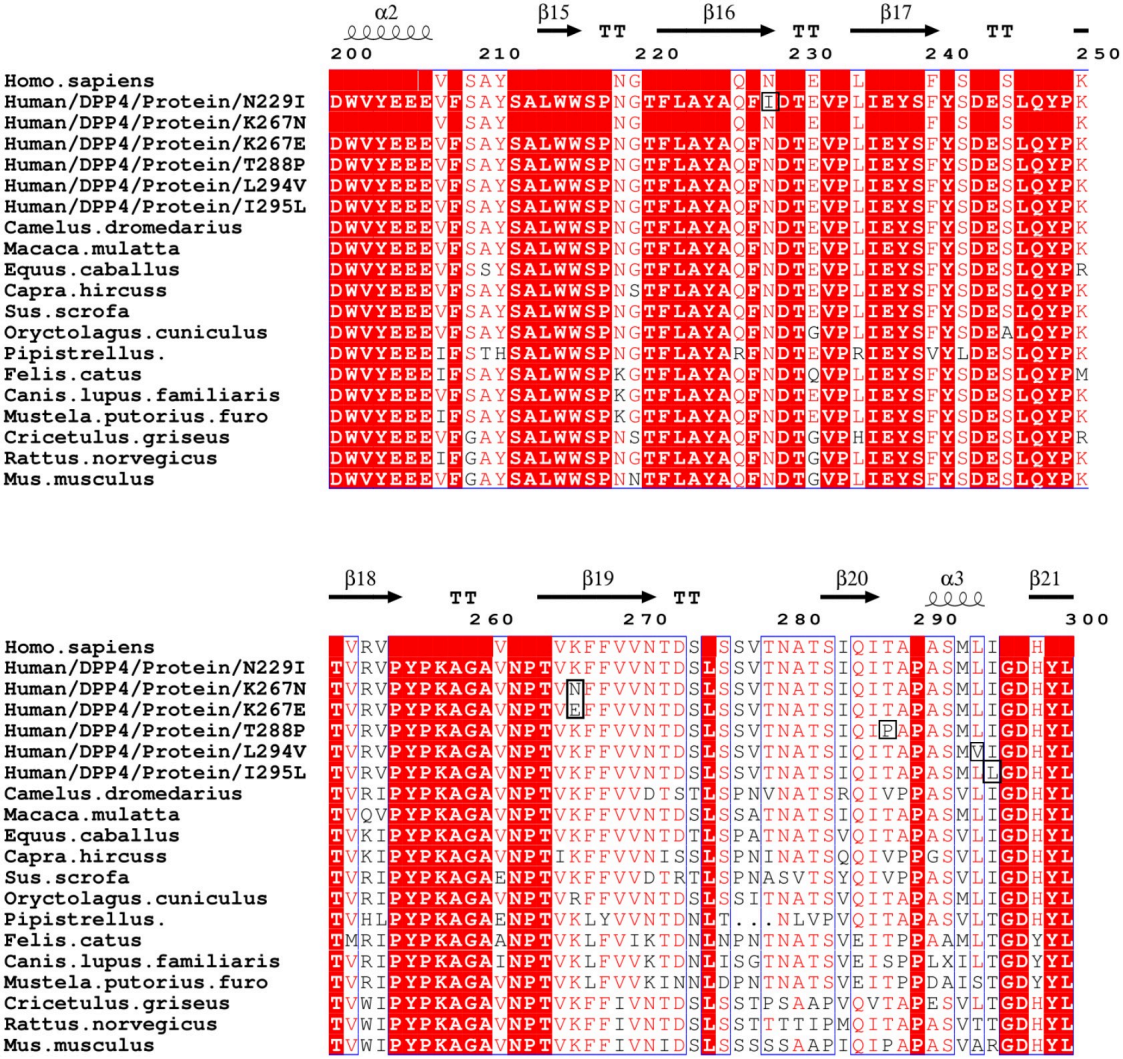
Alignment of human and animal *DPP4* protein sequences by Clustal W. Identical amino acid residues in different species are highlighted with the same residue colour. Mutations identified in this study are highlighted in black.

We aligned study subjects sequences carrying the non-synonymous mutations with MERS-CoV permissive and non-permissive animal species *DPP4* gene to evaluate the interaction residues homology among human identified mutations and wild type protein sequence [*Homo sapiens* (NP_001926.2), *Macaca mulatta* (NP_001034279.2), *Equus caballus* (XP_001494049.2), *Capra hircus* (KF574265.1), *Camelus dromedarius* (AHK13386.1), *Sus scrofa* (NM_214257.1), *Oryctolagus cuniculus* (XP_002712206.1), *Pipistrellus pipistrellus* (AGF80256.1), *Felis catus* (NP_001009838.1), *Canis lupus familiaris* (XP_535933.3), *Mustela putorius furo* (KF574264.1), *Cricetulus griseus* (XP_007608372.1), *Rattus norvegicus* (NP_036921.1), *Mus musculus* (NP_034204.1)]. Curiously, one human study subject carried the same residue as the mouse at the residue number 288 (Pro288 instead of Thr288) (Fig 1).

### Study population characteristics and mutational relevance

Patients carrying synonymous or non-synonymous mutations identified during this study presented a variable serological profile towards MERS-CoV. A seropositive or borderline seropositive profile by the ELISA technique was found in seven participants, three of whom presented a non-synonymous mutation and four a synonymous mutation. However, only two patients with nonsynonymous and synonymous mutations, respectively, have neutralizing antibodies to MERS-CoV. Three patients with a seronegative profile for MERS-CoV presented a non-synonymous mutation at the level of the interaction amino acid residues, the L294V mutation was found in two seronegative patients. The N229I mutation was characterized in a seronegative patient (Table 3).

**Table 3:** Participants serological profile with identified mutations in amino acid residues engaging the interaction of *DPP4* with MERS-CoV.

*Participant ID*	*Nucleotide Mutation (NM_001935*.*4)*	*Amino acid Mutation (NP_001926*.*2)*	*Mutation Type*	*ELISA Results* ^a^ [8]	*Neutralization Results (ppNT + PRNT)*^b^ [8]
**G030**	c.862A>C	p.Thr288Pro	Non-synonymous	Borderline Positive	Negative
**L005**	c.880T>G	p.Leu294Val		Negative	Negative
**L123**	c.884T>A	p.Ile295Leu		Positive	Positive
**L148**	c.801G>C	p.Lys267Asn		Borderline Positive	Negative
**G050**	c.799A>G	p.Lys267Gln		Negative	Negative
**D146**	c.686C>T	p.Asn229>Ile		Negative	Negative
**D196**	c.880T>G	p.Leu294Val		Negative	Negative
**G008**	c.951G>A	p.Arg317 =	Synonymous	Positive	Negative
**G091**	c.951G>A	p.Arg317 =		Positive	Negative
**L057**	c.872T>C	p.Ala291 =		Borderline Positive	Positive
**D002**	c.950G>A	p.Arg317 =		Borderline Positive	Negative

^a^ Optical Density (OD) ratios recommended as cut off for positive (1.1) and borderline (0.8) by the ELISA kit manufacturer.

^b^ Positives defined as ppNT-positive and confirmed by ≥ 90% reduction of plaque counts in PRNT test.

There is no statistical significance according to the Fisher test, between the presence of a mutation, synonymous or non-synonymous, at one of the *DPP4* binding residues with MERS-CoV S1 RBD according to participants sex (Fisher: p-value = 0.29) or the type of exposure (Fisher: p-value = 0.32). The correlation between the serological profile of the participants and the presence of a mutation at *DPP4* binding residues is not significant according to the ELISA test (Fisher: p-value = 0.29) or the presence of neutralizing antibodies (Fisher: p-value = 0.059) (Table 4).

**Table 4:** Statistical significance for association of sex, type of exposure and seropositivity with the presence of a mutation in the receptor *DPP4* binding residues according to the Fisher and Risk Ratio test.

*Characteristics*	*Presence of mutation*	*Absence of mutation*	*Statistical significance with Fisher test*	*Risk Ratio (RR) and Interval of confidence [IC]*
***Sex***				
**Male**	10	66	Test Fisher = 0.2889	3.16 [0.42, 23.42]
**Female**	1	23	Non-significant	
***Exposure groups***				
**Direct exposure groups (Camels herders, Slaughterhouse workers)**	9	56	Test Fisher = 0.32	2,42 [0.55, 10.60]
**Indirect exposure group (General population)**	2	33	Non-significant	
***Serological profile of participants by ELISA assay*** [8]				
**Seronegative**	4	51	Test Fisher = 0.2889	2.13 [0.66, 6.84]
**Seropositive and Borderline seropositive**	7	38	Non-significant	
***Serological profile of neutralisation assay (ppNT + PRNT)*** [8]				
**Seronegative**	9	87	Test Fisher = 0.0588	5.33 [1.67, 17.02]
**Seropositive**	2	2	Non-significant	

### Reliability of protein structure

All prepared *DPP4* structures were verified using MolProbity to generate Ramachandran plot [28], ProSA analysis [30] and Verify 3D [29] before prediction of mutation effect analysis, docking and MD simulation. Ramachandran plot showed 91.2%, 91.5%, 92.3%, 91.7%, 91.7%, 91.6% and 91.5% of *DPP4*-WT, *DPP4*-N229I, *DPP4*-K267N, *DPP4*-K267E, *DPP4*-T288P, *DPP4*-L294V and *DPP4*-I295L residues were respectively in favored regions. However, *DPP4* residues in allowed region were 8.1%, 7.9%, 7.1%, 7.9%, 7.7%, 7.6% and 7.7% of *DPP4*-WT, *DPP4*-N229I, *DPP4*-K267N, *DPP4*-K267E, *DPP4*-T288P, *DPP4*-L294V and *DPP4*-I295L, respectively. The *DPP4* outlier residues were 0.7%, 0.5%, 0.5%, 0.4%, 0.5%, 0.8% and 0.8% of *DPP4*-WT, *DPP4*-N229I, *DPP4*-K267N, *DPP4*-K267E, *DPP4*-T288P, *DPP4*-L294V and *DPP4*-I295L, respectively (S1 Fig). ProSA analysis verified prepared *DPP4* structures and the *z-score* ranging between -10.82 and -10.87 (S1 Table). All tertiary structures of *DPP4* models passed the Verify 3D verification ranging between 89.16% and 94.53% (S1 Table).

To generate a complex protein model of MERS-CoV S1 RBD protein bound to *DPP4* structure via HADDOCK webserver. According to HADDOCK, the top cluster is the most reliable [31]. Thus, the first model of the top cluster was selected for each complex model after verification of HADDOCK score, *z-score*, RMSD, Van der Waals energy, electrostatic energy, desolvation energy, restraints violation energy and buried surface area (S2 Table). Prior to MD simulation, each complex model was submitted first to energy minimization. Reliability of complex models were assessed through energy potential, temperature, pressure and density parameters (S3 Table).

### Prediction of mutation effect through prediction tools

To model the influence of identified *DPP4* mutations on direct binding residues with MERS-CoV S1 RBD protein, we performed a computational analysis on *DPP4* –MERS-CoV S1 RBD protein complex stability using Mutabind2 and DynaMut computational prediction tools. Each prediction tool uses custom calculation parameters linked to the Gibson free energy (ΔΔG) equation, which results in a disparity in the predictive effect of a mutation on PPI. Our results exhibit a destabilizing effect on binding affinity in all mutant complex models using Mutabind2 tool (Table 5). Contrariwise, the effect on protein complex stability and change in folding energy predicted a destabilizing effect by DynaMut upon *DPP4* mutations K267N, L294V and I295L. While a stabilizing effect was predicted following N229I, K267E and T288P mutation in *DPP4* (Table 5).

**Table 5:** Assessment of the effect of human *DPP4* mutations identified among the population study on protein-protein interaction (PPI) using computational prediction tools Mutabind2 and DynaMut.

Mutation	Mutabind 2		DynaMut	
	ΔΔG (Kcal/mol)	Predictive mutation effect	ΔΔG (Kcal/mol)	Predictive mutation effect
**N229I**	0.47	Destabilizing	1.216	Stabilising
**K267N**	1.9	Destabilising	-0.418	Destabilising
**K267E**	3.63	Destabilising	0.414	Stabilising
**T288P**	0.03	Destabilising	0.261	Stabilising
**L294V**	2.0	Destabilising	-0.174	Destabilising
**I295L**	0.94	Destabilising	-0.212	Destabilising

### Molecular dynamics (MD) of *DPP4* –MERS-CoV S1 RBD protein complex wild type and mutant models

#### Root mean square deviation (RMSD)

**Fig 2:**
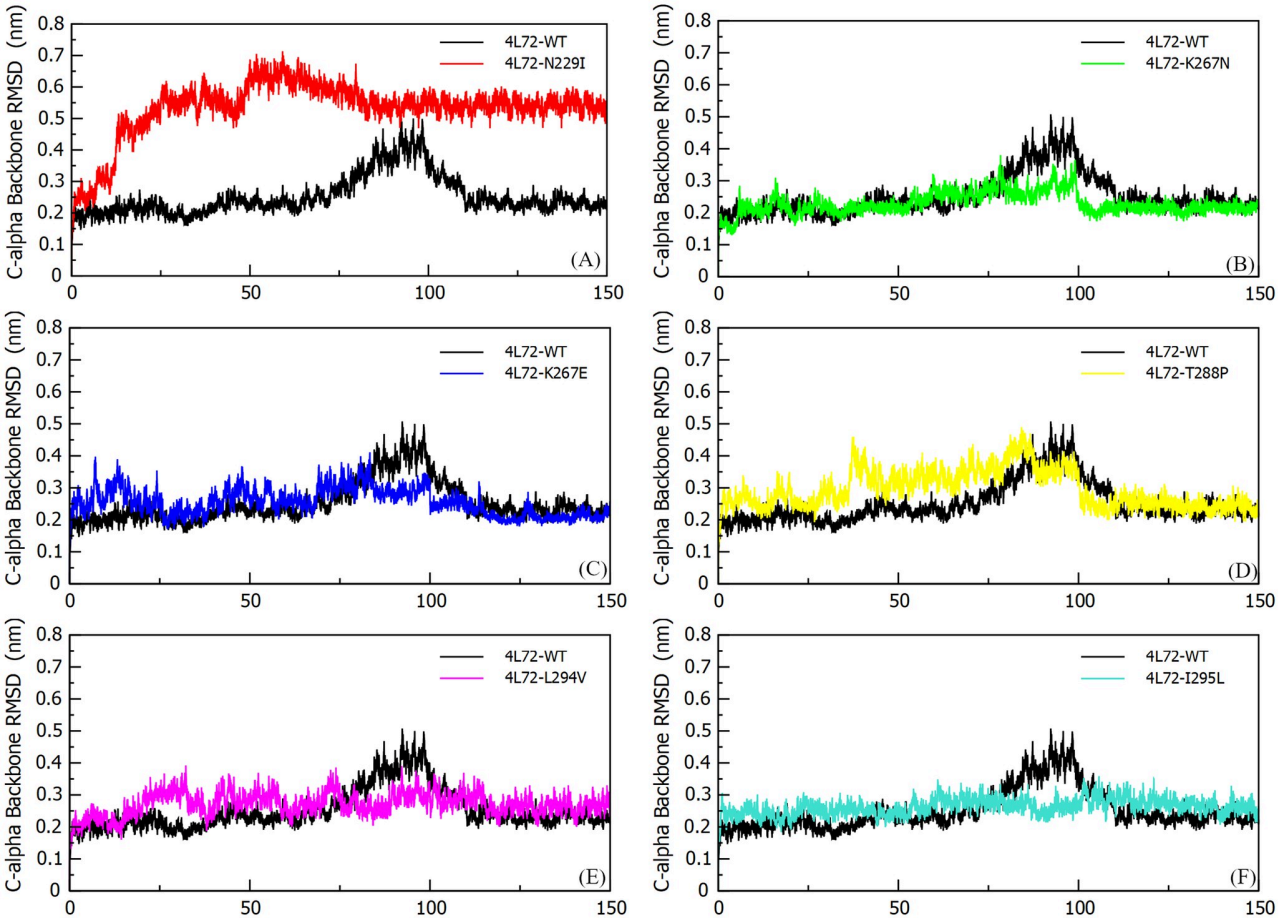
Cα-Backbone root mean square deviation (RMSD) of the human *DPP4* protein in complex with MERS-CoV S1 RBD protein, during 150 ns of the molecular dynamics simulation period. (a) RMSD 4L72-WT vs 4L72-N229I. (b) RMSD 4L72-WT vs 4L72-K267N. (c) RMSD 4L72-WT vs 4L72-K267E. (d) RMSD 4L72-WT vs 4L72-T288P. (e) RMSD 4L72-WT vs 4L72-L294V. (f) RMSD 4L72-WT vs 4L72-I295L.

Trajectory stability of the models conformations during MD simulation was evaluated using RMSD analysis. The average RMSD value of Cα-Backbone of *DPP4* –MERS-CoV S1 RBD protein complex wild type model (4L72-WT) was 0.251 (+/- 0.062) nm, and was similar to the complex mutant models 4L72-K267N (0.228 +/- 0.033 nm), 4L72-K267E (0.254 +/- 0.042 nm), 4L72-T288P (0.295 +/- 0.059 nm), 4L72-L294V (0.271 +/- 0.035 nm) and 4L72-I295L (0.262 +/- 0.025 nm). These mutations sustained evidence of gained conformational stability indicating a balanced system. Inversely, the mutant model 4L72-N229I presented a two-fold increase in average RMSD value in comparison to 4L72-WT model (0.532 +/- 0.089 nm) highlighting a conformational stability loss. Cα-Backbone RMSD of *DPP4* –MERS-CoV S1 RBD protein complex of wild type against each mutant model were plotted in Fig 2. The average Cα-Backbone RMSD value of *DPP4* wild type model (*DPP4*-WT) was 0.2707 (+/- 0.059) nm, and was similar to *DPP4* mutant models, sustaining a conformational stability of the protein, with an average RMSD value for the following models *DPP4*-K267N, *DPP4*-K267E, *DPP4*-T288P, *DPP4*-L294V and *DPP4*-I295L respectively of 0.25 (+/- 0.02) nm, 0.25 (+/- 0.03) nm, 0.28 (+/- 0.04) nm, 0.27 (+/- 0.02) nm and 0.27 (+/-0.02) nm. Unlike *DPP4*-N229I model returned a Cα-Backbone RMSD average value of 0.473 (+/- 0.080) nm indicating a conformational stability loss (S2 Fig). Likewise, the average Cα-Backbone RMSD value of MERS-CoV S1 RBD in wild type model was 0.299 (+/- 0.09) nm, while average RMSD value of MERS-CoV S1 RBD in 4L72-N229I model was 0.841 (+/-0.14) (S2 Fig). Yet, a slightly higher RMSD average value MERS-CoV S1 RBD in the following models 4L72-K267N (0.35 +/- 0.06 nm), 4L72-K267E (0.44 +/- 0.06 nm), 4L72-T288P (0.49 +/- 0.08 nm), 4L72-L294V (0.41 +/- 0.06 nm) and 4L72-I295L (0.40 +/- 0.05 nm) in comparison to wild type structure (S2 Fig).

#### Root mean square fluctuation (RMSF)

**Fig 3:**
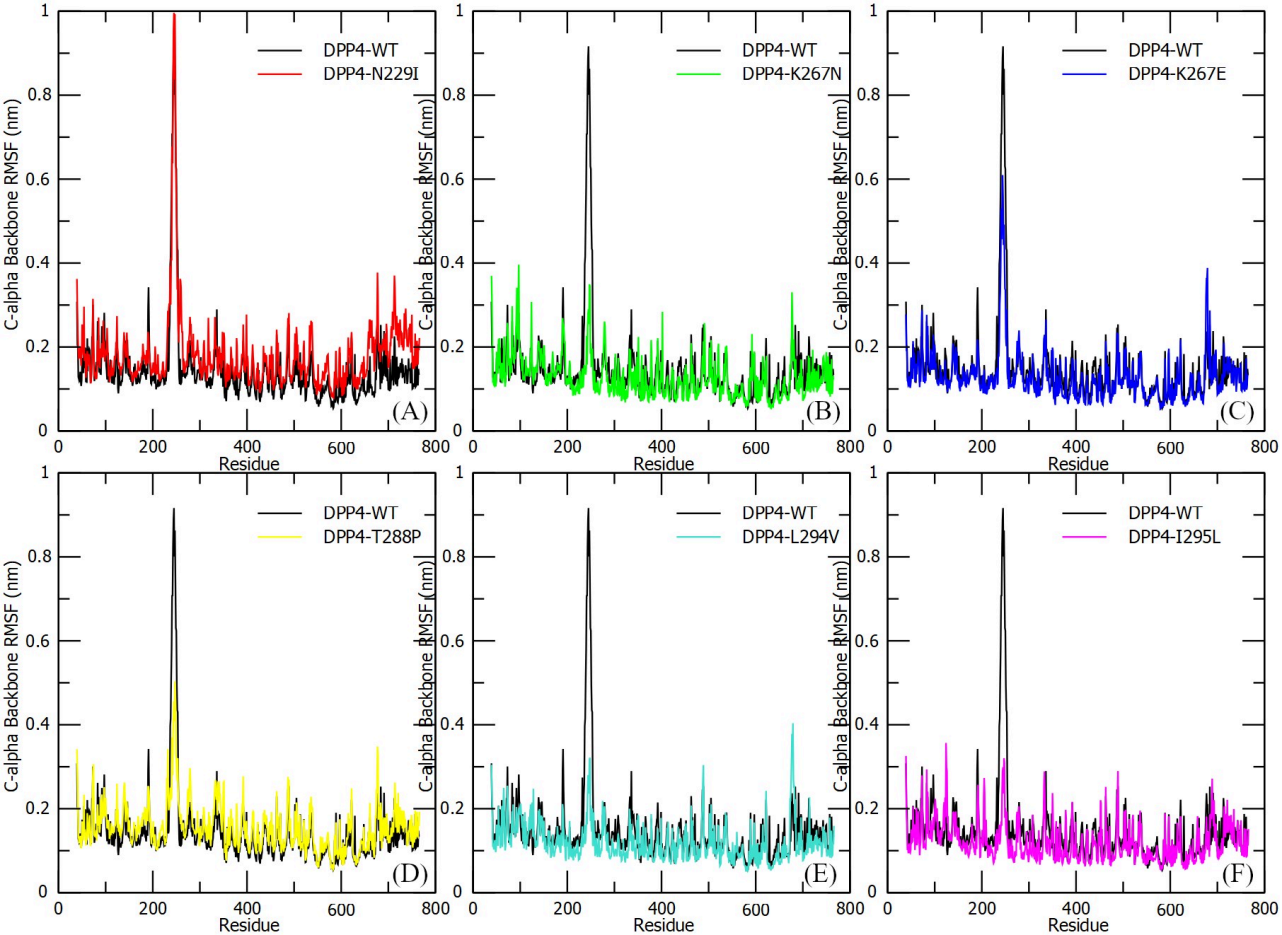
Cα-Backbone root mean square fluctuation (RMSF) of the human *DPP4* protein in complex with MERS-CoV S1 RBD protein, during 150 ns of the molecular dynamics simulation period. (a) RMSF 4L72-WT vs 4L72-N229I. (b) RMSF 4L72-WT vs 4L72-K267N. (c) RMSF 4L72-WT vs 4L72-K267E. (d) RMSF 4L72-WT vs 4L72-T288P. (e) RMSF 4L72-WT vs 4L72-L294V. (f) RMSF 4L72-WT vs 4L72-I295L.

*DPP4* protein residual flexibility and local movement were characterized using Cα-Backbone RMSF in wild type and mutant models, and plotted against residue position numbers (Fig 3). A large fluctuation within the *DPP4* receptor binding motif (RBM) to the MERS-CoV S1 RBD protein, located between residues 119 and 354 at blade IV and V of the antiparallel β-sheet of the *DPP4* protein were observed (Fig 3). The highest RMSF value within the *DPP4* RBM was witnessed for 4L72-WT model at 0.91 nm. While, the *DPP4* mutant models were less flexible than the 4L72-WT model with the highest RMSF value within *DPP4* RBM was 0.34 nm, 0.61 nm, 0.5 nm, 0.4 nm and 0.35 nm respectively for mutant models 4L72-K267N, 4L72-K267E, 4L72-T288P, 4L72-L294V and 4L72-I295L (Fig 3B–3F). Although, the *DPP4* mutant model carrying N229I mutation inferred an increase of conformation flexibility linked to a higher fluctuation of *DPP4* RBM with the highest RMSF value of 0.99 nm compared to 4L72-WT (Fig 3A). Whereas MERS-CoV S1 RBD protein highlighted a similar overall residual flexibility in wild type and mutant complex models, key binding residues returned comparable RMSF values in wild type and mutant complex models while emphasizing a higher fluctuation in protein C-terminal and N-terminal regions (S2 Fig).

#### Radius of gyration (Rg)

**Fig 4:**
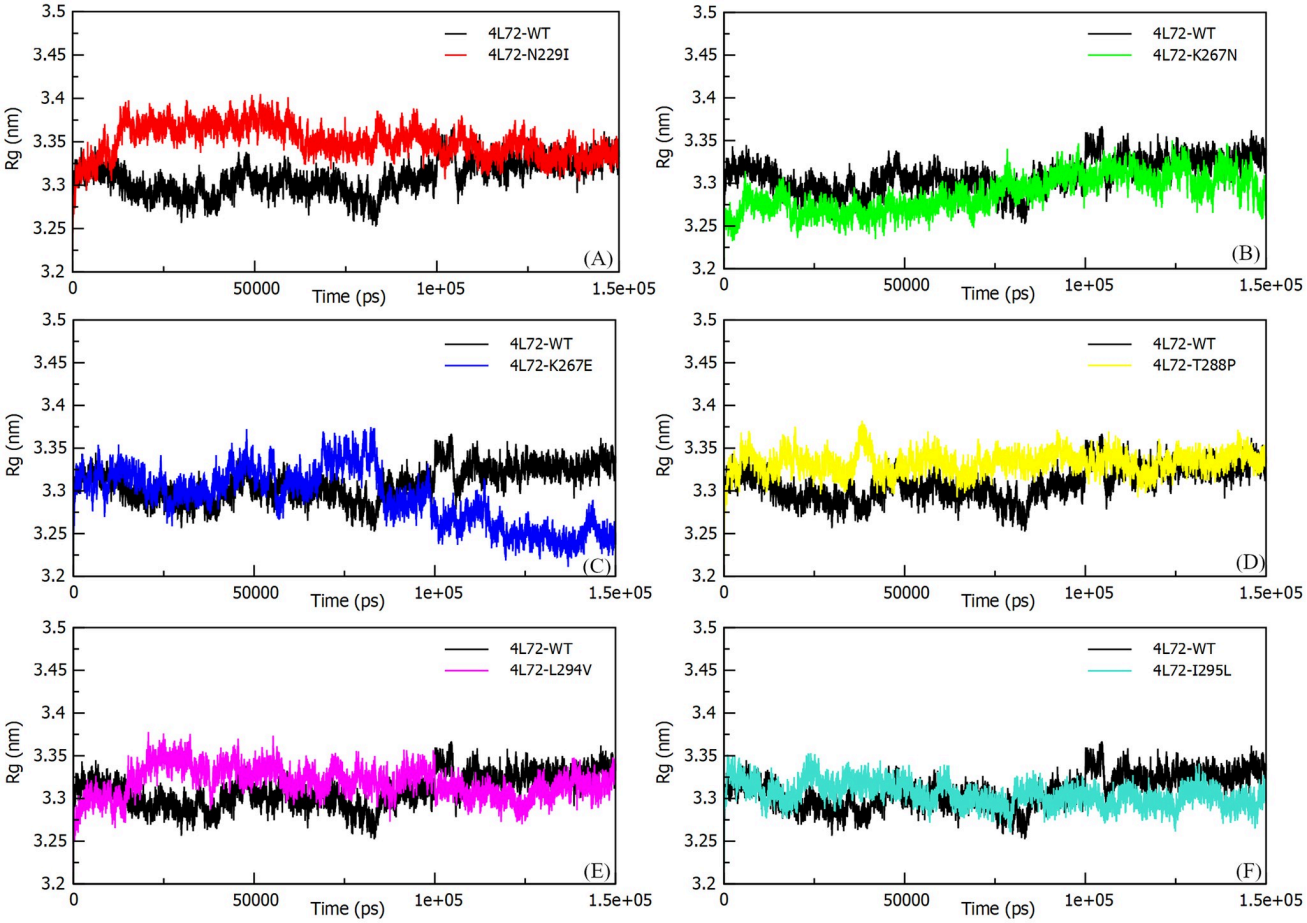
Cα-Backbone Radius of gyration (Rg) of the human *DPP4* protein in complex with MERS-CoV S1 RBD protein, during 150 ns of the molecular dynamics simulation period. (a) Rg 4L72-WT vs 4L72-N229I. (b) Rg 4L72-WT vs 4L72-K267N. (c) Rg 4L72-WT vs 4L72-K267E. (d) Rg 4L72-WT vs 4L72-T288P. (e) Rg 4L72-WT vs 4L72-L294V. (f) Rg 4L72-WT vs 4L72-I295L.

The PPI compactness characterizing folding, shape and stability of the dynamic complex structure over time was evaluated by measuring the Rg of the complex as well as its individual component, i.e: *DPP4* and MERS-CoV S1 RBD. Small Rg values designate a compact protein structure while higher Rg values designate loose protein structure. Mutant models (4L72-K267N, 4L72-K267E, 4L72-T288P, 4L72-L294V and 4L72-I295L) showed a relative constant Rg values neighboring wild type (4L72-WT) model Rg values through MD simulation (Fig 4B–4F). Whereas, 4L72-N229I model highlights an abrupt increase fluctuation of Rg values unlike 4L72-WT after 11 ns of MD simulation before reaching a stability of complex structure compactness (Fig 4A). However, the compactness of *DPP4* and MERS-CoV S1 RBD individually highlighted a stable compactness of proteins structure throughout MD simulation, as Rg mean values were comparable to wild type model (S3 Fig).

#### Solvent accessible surface area (SASA)

**Fig 5:**
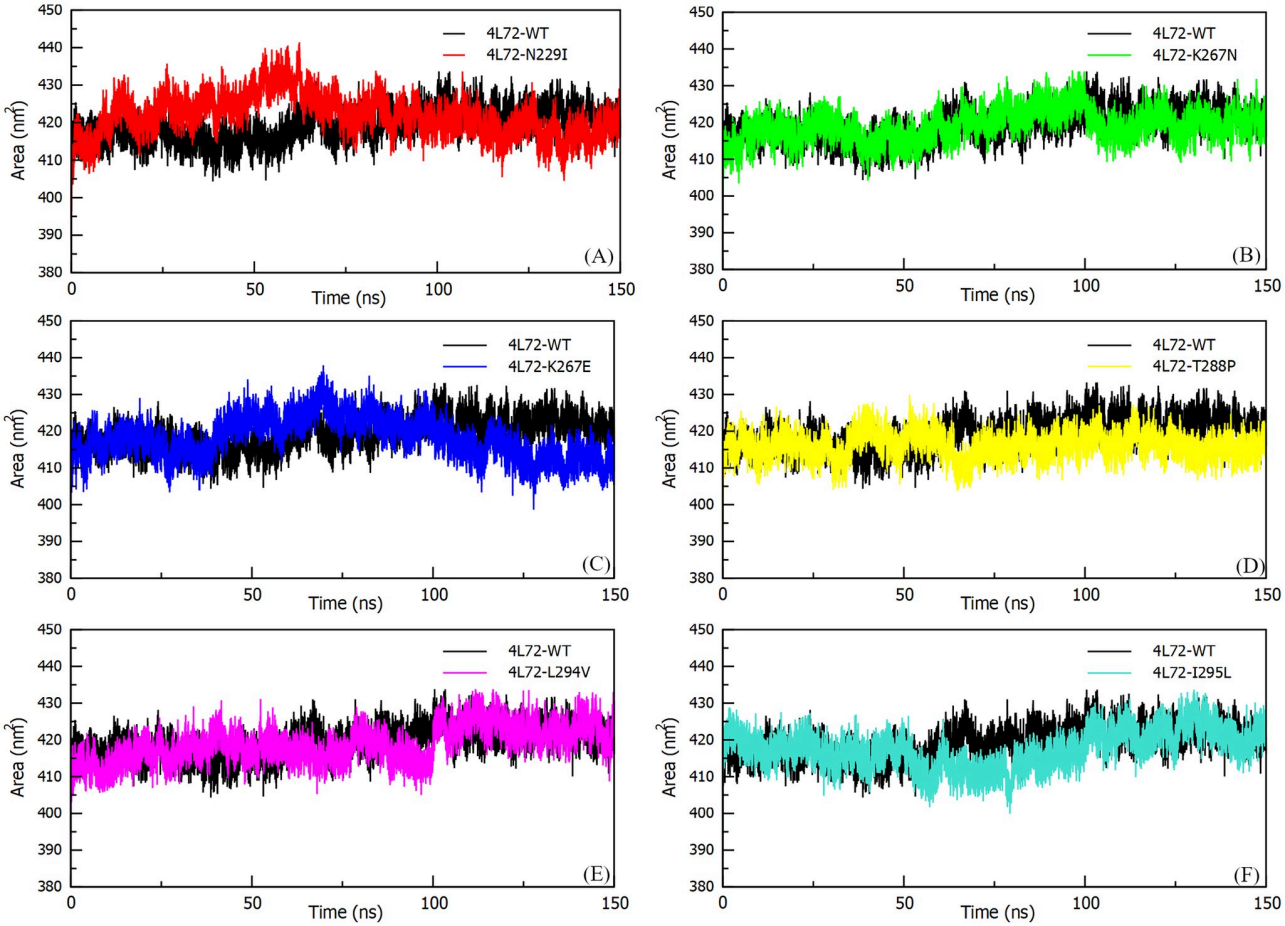
Solvent accessible surface area (SASA) between MERS-CoV S1 RBD protein and human *DPP4* wild and mutant types during 150 ns of the molecular dynamics simulation period. (a) Rg 4L72-WT vs 4L72-N229I. (b) Rg 4L72-WT vs 4L72-K267N. (c) *Rg 4L72-WT vs 4L72-K267E*. (d) *Rg 4L72-WT vs 4L72-T288P*. (e) Rg 4L72-WT vs 4L72-L294V. (f) Rg 4L72-WT vs 4L72-I295L.

The surface area of a biomolecule interacting with the solvent molecules was evaluated with SASA parameter to determine its conformational stability in an aqueous medium. Since *DPP4* binding residues to MERS-CoV S1 RBD protein are present at the surface of the protein, thus the presence of mutations at this level induce an accessibility conservation of solvent to protein surface area during MD simulation (Fig 5B–5F). Still, the N229I mutation abruptly seems to fluctuate significantly between 40–60 ns. After 57 ns simulation, 4L72-N299I model reaches the highest SASA value of 440 nm^2^ while 4L72-WT model reaches the lowest SASA value of 407 nm^2^ during MD simulation. Nevertheless, both models (WT and N229I) stabilize at the same average SASA value of 425 nm^2^ between 60 ns and 150 ns (Fig 5A). Regarding complex model components, wild type and mutant *DPP4* and MERS-CoV S1 RBD protein structures maintained respectively a similar SASA average value throughout the MD simulation, with an abrupt fluctuation between 40 and 60 ns for *DPP4*-N229I structure prior stabilizing at SASA average value of 338 nm^2^ (S3 Fig).

#### Hydrogen bonds (H-bonds)

**Fig 6:**
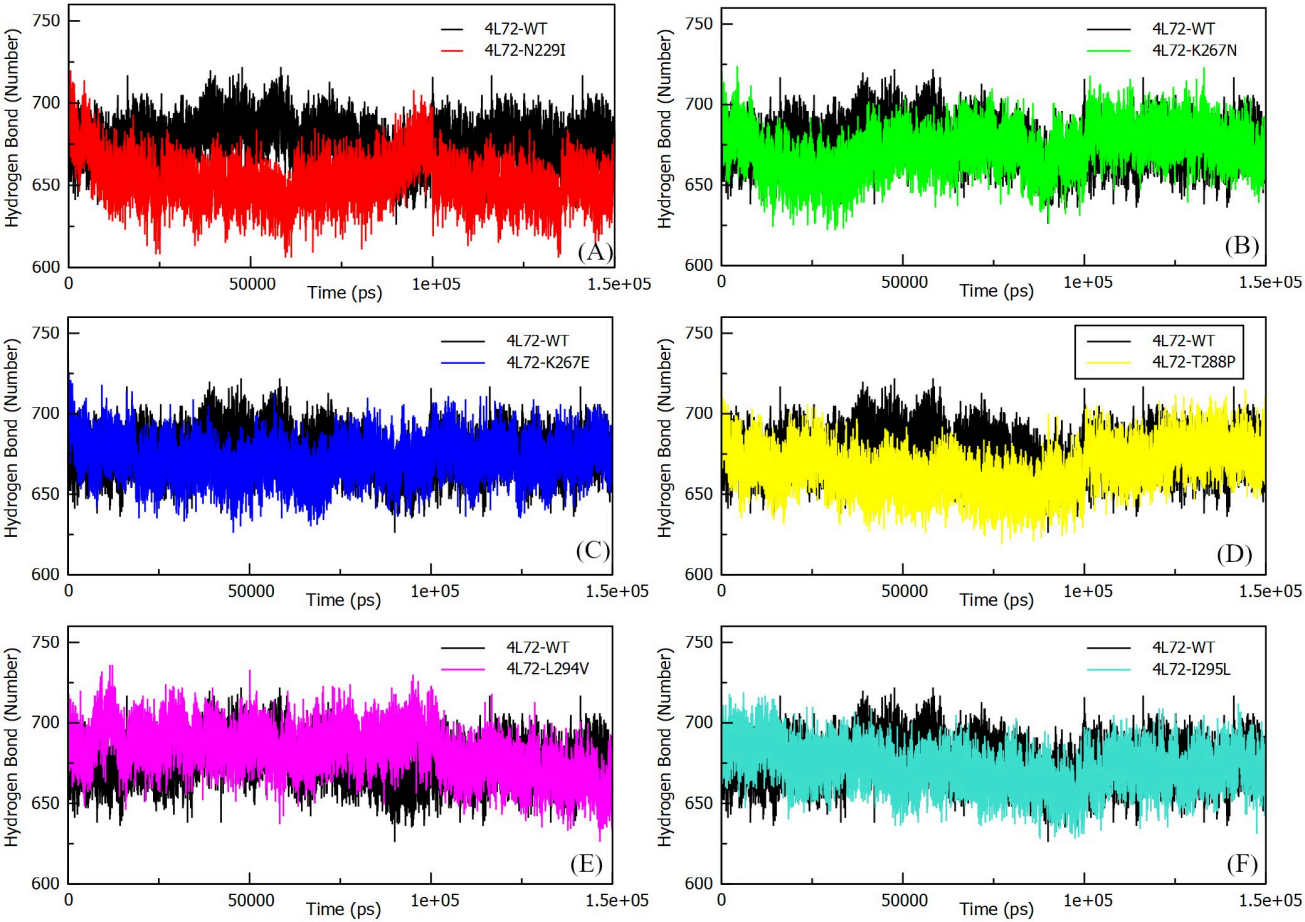
Total number of hydrogen bonds interactions between MERS-CoV S1 RBD protein and human *DPP4* wild and mutant types during 150 ns of the molecular dynamics simulation period. (a) H-bond 4L72-WT vs 4L72-N229I. (b) H-bond 4L72-WT vs 4L72-K267N. (c) H-bond 4L72-WT vs 4L72-K267E. (d) H-bond 4L72-WT vs 4L72-T288P. (e) H-bond 4L72-WT vs 4L72-L294V. (f) H-bond 4L72-WT vs 4L72-I295L.

**Fig 7:**
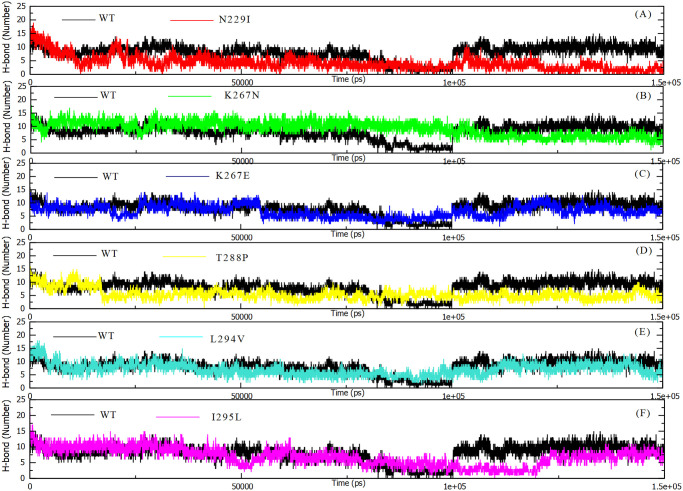
Number of hydrogen bonds at the interface level of interacting residues of MERS-CoV S1 RBD protein and human *DPP4* wild and mutant types during 150 ns of the molecular dynamics simulation period. (a) H-bond 4L72-WT vs 4L72-N229I. (b) H-bond 4L72-WT vs 4L72-K267N. (c) H-bond 4L72-WT vs 4L72-K267E. (d) H-bond 4L72-WT vs 4L72-T288P. (e) H-bond 4L72-WT vs 4L72-L294V. (f) H-bond 4L72-WT vs 4L72-I295L.

Hydrogen bonds induce formation of secondary and tertiary protein structure. An increase of hydrogen numbers infer stronger PPI interactions. MD simulation of mutant models highlighted a notable increase in hydrogen interaction at the level of the complex formed during the interaction of *DPP4* with MERS-CoV S1 RBD protein (Fig 6). Thus, the increase in hydrogen bonding implies a stability of the bond between the two proteins participating in the formation of the complex overtime regardless of conformation change in case of 4L72-N229I model. Yet, the average number of hydrogen bonds present at the interface between *DPP4* and MERS-CoV S1 RBD during MD simulation was 8.012 +/- 2.672 in wild type model, while N229I mutant model returned the lowest h-bonds number (4.178 +/- 2.622) in 150 ns MD simulation reaching a null h-bonds at the interface of both proteins at 150 ns (Fig 7). Yet, other mutant models highlighted similar or stronger binding of complex protein as number of h-bonds present between their interfaces and was comparable or higher than wild type model (Fig 7).

#### Principal component analysis

**Fig 8:**
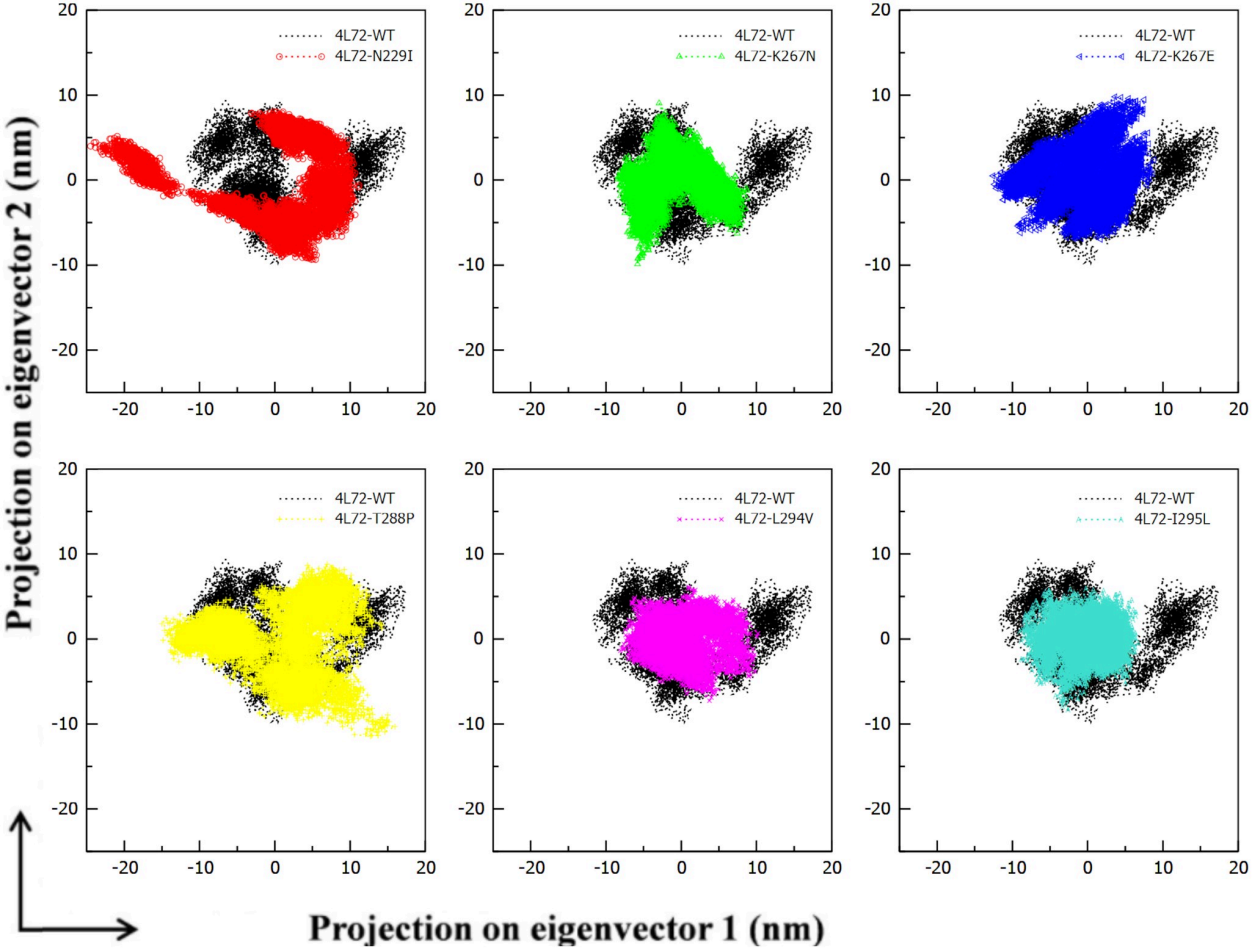
Principal component analysis 2-D projection MERS-CoV S1 RBD in complex with *DPP4* wild and mutant type in phase space along first two principal eigenvectors. (a) RMSD 4L72-WT vs 4L72-N229I. (b) RMSD 4L72-WT vs 4L72-K267N. (c) RMSD 4L72-WT vs 4L72-K267E. (d) RMSD 4L72-WT vs 4L72-T288P. (e) RMSD 4L72-WT vs 4L72-L294V. (f) RMSD 4L72-WT vs 4L72-I295L.

Principal components analysis (PCA) extracts the dominant modes of motion and magnitude in a molecule from trajectory of MD simulation resulting a matrix of eigenvectors and a set of associated eigenvalues that combined highlights respectively principal component (PC) and amplitude of local motion within a protein. Thus, to understand the dynamics a the complex we have generated a 2-D projection plot relaying on two PC coordinates projecting the overall dataset into manageable compact dimension appropriate for 2-D plotting. Fig 8 highlights the projection of two eigenvectors for *DPP4* –MERS-CoV S1 RBD complex models. The complex with less phase space and a stable cluster indicated a more stable structure, whereas the complex with greater space and a non-stable cluster denoted a less stable structure. Therefore, complex models 4L72-K267N, 4L72-K267E, 4L72-L294V and 4L72-I295L were regarded as more stable structures compared to 4L72-WT complex model. Whereas, 4L72-T288P complex showed similar structural stability as 4L72-WT complex. Yet, 4L72-N229I denoted a less stable complex than 4L72-WT complex (Fig 8).

#### Local structural changes

**Fig 9:**
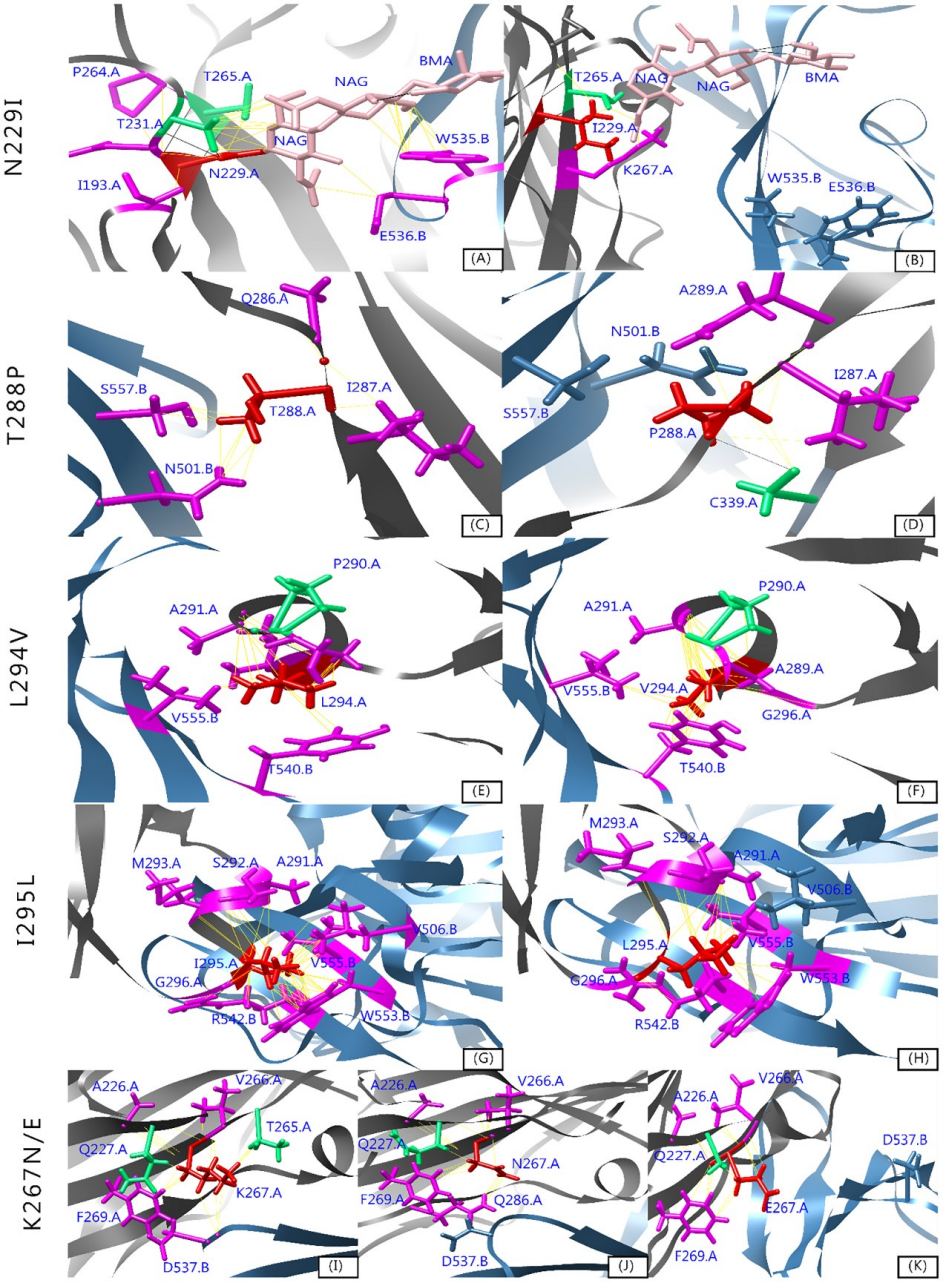
Structural model of wild and mutant 4L72 models after 150 ns MD simulation. Residues substituted are marked in red, residues involved in hydrophobic interactions are indicated in magenta, residues engaging simultaneous hydrogen bonding and hydrophobic contacts are indicated in green. *DPP4* (chain A) and MERS-CoV S1 RBD protein (chain B) are dyed respectively with grey and blue. Ligands (NAG, BMA) are highlighted in pink. Hydrogen bonds are shown in black line and hydrophobic/ionic contacts are shown in yellow line.

Through the MD simulation, a structural comparison between wild type and mutant 4L72 models at different time scales, highlighted a structural conservation of the protein complex models at the beginning of MD simulation (t = 0 ns) (S4 Fig). However, throughout the simulation the 4L72-WT sustains a structural stability in contrast with the 4L72 mutant models highlighting structural changes and folding specially in the MERS-CoV S1 RBD protein (S4 Fig). Then, using the three dimensional structure of the complex models, we compared local structural changes induced by these introduced mutations (N229I, K267N, K267E, T288P, L294V, I295L) upon MD simulation using Chimera 1.15 software [35] (Fig 9). Each of these *DPP4* key binding residues interact with MERS-CoV S1 RBD protein via hydrophobic bonds. Thus, the presence of a mutation on these locations induce a loss of existing bonding for all mutant models, except for L294V and I295L, responsible for the local structural changes during the MD simulation (Fig 9). Upon *DPP4* N229I mutation, this residue lost an h-bond with T231 and two hydrophobic bonds with I193 and P264. Thus, bringing *DPP4* blade IV domain closer by 0.8 Å and looseness of *DPP4* blade V domain by 0.4 Å. However, I229 lost a hydrophobic binding with NAG-NAG-BMA ligand inferring a loss of interaction with MERS-CoV S1 RBD binding residues W535 and E536. *DPP4* residue 288 is located between blade IV and V of the protein. *DPP4* T288P mutation brought the blade IV domain closer to the structure by 1.1 Å, gaining a hydrogen bond with C339. Yet *DPP4* T228P mutation provoked a loss of hydrophobic binding with MERS-CoV S1 RBD binding residues N501 and S557. *DPP4* residue 294 is located at the end of the short helix α3 in blade IV. *DPP4* L294V marks a gain of hydrophobic bond within *DPP4* structure with G296, bringing blade IV domain closer to structure by 0.3 Å. However, no effect have been observed on hydrophobic binding with MERS-CoV S1 RBD binding residues T540 and W555. Regarding K267N and K267E mutation, *DPP4* 267 residue lost a hydrogen bond with T265 prompting a narrower blade IV and V by 0.3 Å and 0.1 Å respectively. This structural effect point toward a higher compact complex structure upon *DPP4* K267 mutation.

## Discussion

In the present study, we report our results by addressing the effect of genetic variability of *DPP4* receptor binding residues with MERS-CoV S1 RBD protein in a human population with a significant exposure risk of infection with MERS-CoV via dromedaries. Our study is the first to address this concern by selecting a study population from a human population in the southern regions of Morocco, highly exposed to camels, with a neutralizing antibody seroprevalence of 0.83% [8]. The mutations K267E and K267N identified in this study were previously described and listed in genomic databases with very low mutation allelic frequencies. Yet, the mutations N229I, T288P, A291 =, L294V, I295L and R317 = carried by the study participants are described for the first time in the present work.

The presence of the non-synonymous mutation L294V and I295L appear not to disrupt *DPP4* and MERS-CoV S1 RBD binding affinity, and to safeguard the ability of the virus to complete its replicative viral cycle at the cellular level. The amino acids Isoleucine (Ile), Leucine (Leu) and Valine (Val) holds similar physicochemical properties of a non-polar nature with an aliphatic side chain, which could explain this binding conservation of *DPP4* and MERS-CoV. As *DPP4* L294V mutation favored a gain of two hydrophobic bonds with A289 and G296 bringing *DPP4* blade IV domain closer by 0.3 Å. While *DPP4* I295L maintained hydrophobic interaction within *DPP4* protein, thus no structural changes were observed in critical *DPP4* blade IV and V binding domain. However, *DPP4* residue 294 has been described as a critical residue in MERS-CoV cellular permissiveness in chimeric *mDPP4* carrying A294L [36, 37]. Interestingly, we have reported a novel *DPP4* T288P mutation that is common in wild type murine *DPP4* (*mDPP4*) protein. Murine species have been proven non-permissive to MERS-CoV, presenting a P288 and four different residues at glycosylation sites [36]. As *mDPP4* P288T mutation had no effect on MERS-CoV cellular permissiveness; therefore, *DPP4* residue 288 is not critical on human permissiveness to MERS-CoV and infection outcome [36, 37]. Remarkably, a computational study described *DPP4* 288 residue as critical inferring a significant flexibility on *DPP4* protein without disturbing the binding standing conformation of MERS-CoV S1 RBD after docking with *DPP4* [12]. These findings are in accordance with the effect of the sole T288P mutation on *DPP4* –MERS-CoV S1 RBD complex stability described in the present study highlighting a narrower *DPP4* blade IV by (1.1 Å) gaining an hydrogen bond with *DPP4* C339. Yet a loss of hydrophobic bonds with MERS-CoV S1 RBD binding residues N501 and S557 did not disrupt significantly complex compactness as number of hydrogen bonds present at *DPP4* –MERS-CoV S1 RBD interface level were of 5 (+/- 2) hydrogen bonds in last 50 ns of MD simulation.

In order to evaluate the effect of these *DPP4* non-synonymous mutations, a computational analysis by molecular modeling was done to predict their impacts on *DPP4* and MERS-CoV S1 RBD protein binding affinity. Surprisingly, *DPP4* protein carrying one of the identified mutations (K267N, K267E, T288P, L294V and I295L) inducing a more stable structural conformation of the complex mainly linked to a decrease in amino acid residues fluctuation of the *DPP4* RBM located in the critical regions of blade IV and V of the antiparallel β-sheet of the *DPP4* protein structure. The gain in stability of the complex carrying these mutations is related to a narrower *DPP4* blade IV domain due to a gain or loss of a hydrogen bond of key binding residues within *DPP4* structure in each complex model. Thus, a slight increase of compactness and hydrogen interactions within these complex models was perceived in contrast to wild type *DPP4* –MERS-CoV S1 RBD complex model, sustaining a compact complex structure with an average of 5 (+/- 2) hydrogen bonds between *DPP4* –MES-CoV S1 RBD complex interfaces in last 50 ns of MD simulation. Thus, the presence of one of these five mutations in humans could be associated with a more efficient attachment of MERS-CoV to cells carrying the *DPP4* receptor and consequently a probable increase in the capacity of MERS-CoV to replicate in human cells. A contradicting effect of *DPP4* K267N and K267E mutation was described in a study [38] through *in-vitro* cellular modeling. In fact, the presence of asparagine or glycine residues at position 267 instead of lysine residue seems to reduce MERS-CoV infectious ability by repressing virus cellular entry. The N229 residue, via the monosaccharide N-acetylglucosamine (NAG), interact with the amino acids W535 and E536 of MERS-CoV S1 RBD protein warranting the PPI of the complex [13, 14]. However, the residual N229I substitution favored *DPP4* RBM fluctuation, inducing a conformational change and loss of compactness due to a loss of binding of I229 with NAG ligand mediating the binding with MERS-CoV S1 RBD protein. Unlike the wild type complex, N229I mutation prompted a narrower *DPP4* blade IV domain by 0.8 Å and a relaxed blade V domain by 0.4 Å characterized with a loss a hydrogen bond with T231 and two hydrophobic bonds with I193 and P264. This mutation inferred a loss of all hydrogen bonds between the interfaces of *DPP4* –MERS-CoV S1 RBD complex in last 20 ns of MD simulation that could cause a critical destabilization of the interaction between *DPP4* and the MERS-CoV S1 RBD protein, involving a viral replication attenuation.

Interestingly, the assessment of estimated protein-protein relative binding affinity via mutation prediction tools, Mutabind2 and DyanMut, returned divergent Gibson free energy for *DPP4* –MERS-CoV S1 RBD complex in contrast to MD simulation approach. The disparity of results of both computational approaches should account for the nature of the algorithm; MD simulation is a rigorous accurate method whereas ΔΔG prediction tools are non-rigorous throughput methods [39]. The occurrence of a non-synonymous mutation could induce a change in the physicochemical properties of the three-dimensional structure, which could explain this gain or loss of structural stability between *DPP4* and MERS-CoV S1 RBD advanced by our computational approach. Although, *in-vitro* cellular modelling would be advantageous to acquire a broad overview of the effect of these mutations.

*TMPRSS2* enzyme and tetraspanin *CD9* have been exhaustively implicated in their functional role of MERS-CoV cellular attachment and entry [40, 41]. However, it is still unknown whether these enzymes can maintain a cellular entry in case of the presence of a mutation in the *DPP4* binding domain to MERS-CoV. It has also been described that the occurrence of non-synonymous mutations on *ACE-2* protein might disrupt the binding affinity to the novel emergent SARS-CoV-2 through computational approach [42]. There is a serious need to carry out a genomic characterization of the *DPP4* receptor in human population at a large scale, of different ethnicity to get a broader landscape of non-synonymous mutations in *DPP4* binding residues to MERS-CoV. Thence, it will complete our understanding of *DPP4* inter-human genetic variability potential effect on MERS-CoV restricted circulation in some geographic areas.

## Conclusion

In summary, the study of inter-human *DPP4* genomic variability is of great interest in understanding the degree of severity of MERS-CoV in humans that could be associated with the origin of sporadic human cases identified mainly in west Asia. Thus, our computational approach based on the crystallographic structure of *DPP4*—MERS-CoV S1 RBD protein complex highlights a possible increase in the binding affinity between the two proteins in the presence of mutations (K267N, K267E, T288P, L294V, and I295L) and loss of affinity due to the N229I mutation. The latter could play a key role in the stability of the host-virus interaction since it is mediated by the monosaccharide NAG. This study could guide current therapeutic approaches to face the adversities that MERS-CoV presents to global public health.

## Supporting information

S1 Fig Ramachandran plots of *DPP4* structure upon mutagenesis using MolProbity web server. (a) *DPP4*-WT, (b) *DPP4*-N229I, (c) *DPP4*-K267N, (d) *DPP4*-K267E, (e) *DPP4*-T288P, (f) *DPP4*-L294V and (g) *DPP4*-I295L.
(TIF)

S2 Fig Cα-Backbone root mean square deviation (RMSD) and fluctuation of individual component *DPP4* and MERS-CoV S1 RBD. (a) Cα-Backbone root mean square deviation (RMSD) of the human *DPP4* protein during 150 ns of the molecular dynamics simulation period. (b) Cα-Backbone root mean square deviation (RMSD) of MERS-CoV S1 RBD protein during 150 ns of the molecular dynamics simulation period. (c) Cα-Backbone root mean square fluctuation (RMSF) of MERS-CoV S1 RBD protein during 150 ns of the molecular dynamics simulation period.
(TIF)

S3 Fig Cα-Backbone rayon of gyration (Rg) and solvent accessible surface area (SASA) of individual component *DPP4* and MERS-CoV S1 RBD. (a) Cα-Backbone Radius of gyration (Rg) of the human *DPP4* protein during 150 ns of the molecular dynamics simulation period. (b) Cα-Backbone Radius of gyration (Rg) of MERS-CoV S1 RBD protein during 150 ns of the molecular dynamics simulation period. (c) Solvent accessible surface area (SASA) of the human *DPP4* protein during 150 ns of the molecular dynamics simulation period. (d) Solvent accessible surface area (SASA) of the MERS-CoV S1 RBD protein during 150 ns of the molecular dynamics simulation period.
(TIF)

S4 Fig Snapshots of wild type and mutant 4L72 models conformation at each 50 ns simulation time step during MD simulation. *DPP4* and MERS-CoV S protein are dyed respectively with grey and blue. Local structural changes during MD simulation in *DPP4* blade IV and V were highlighted in a red square, while local structural changes during MD simulation in MERS-CoV S1 RBD were highlighted in a red circle.
(TIF)

S1 Table *DPP4* structure model validation values using ProSA and Verify 3D web server. (DOCX)

S2 Table Docking validation values of *DPP4* –MERS-CoV S1 RBD complex structure model using HADDOCK 2.4 webserver. (DOCX)

S3 Table Summary of *DPP4* –MERS-CoV S1 RBD complex structure validation parameters in prior molecular dynamics simulation production. (DOCX)

S4 Table Molecular dynamics analysis parameters (RMSD, RMSF, Rg, SASA and H-bond) mean values and standard deviation for each complex model and individual component, i.e: *DPP4* and MERS-CoV S1 RBD protein. (DOCX)
