# Supplementary figures and images for: Effect of identified non-synonymous mutations in DPP4 receptor binding residues among highly exposed human population in Morocco to MERS-CoV through computational approach
Source: PLoS One. 2021 Oct 14;16(10):e0258750. doi: 10.1371/journal.pone.0258750 (PMC8516309; doi:10.1371/journal.pone.0258750)

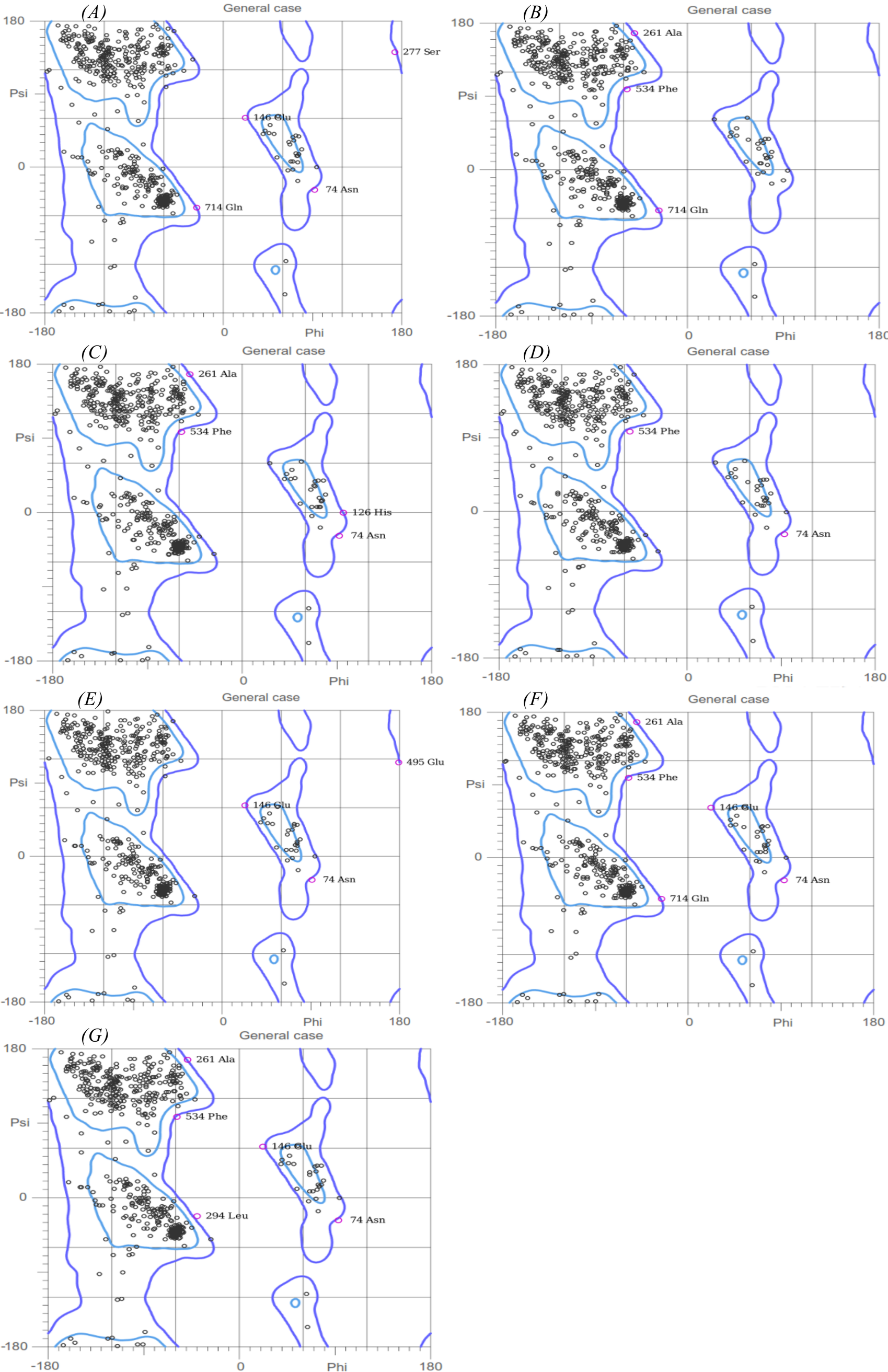

Supplement: S1 Fig — (a) DPP4-WT, (b) DPP4-N229I, (c) DPP4-K267N, (d) DPP4-K267E, (e) DPP4-T288P, (f) DPP4-L294V and (g) DPP4-I295L. (TIF) [file pone.0258750.s001.tif]

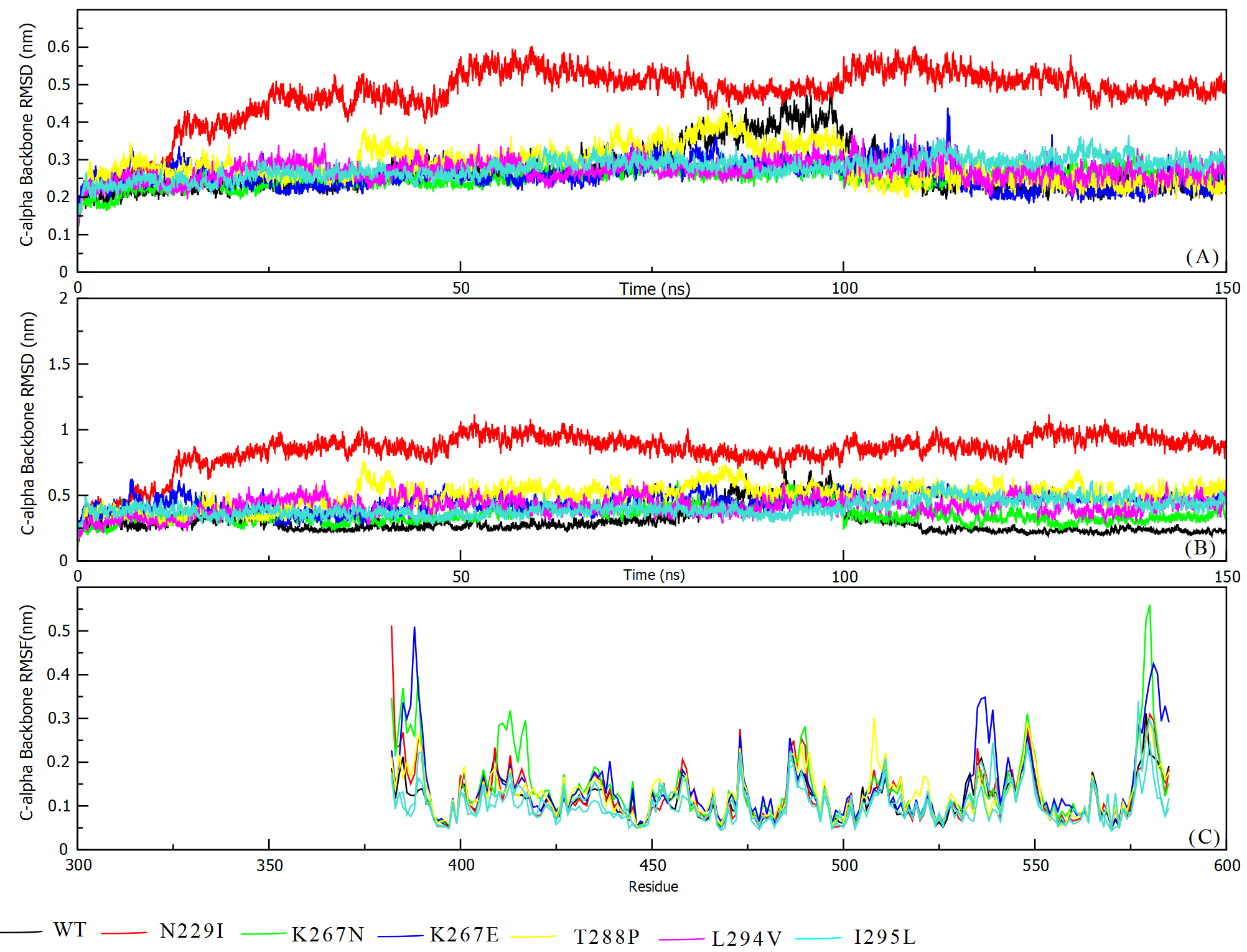

Supplement: S2 Fig — (a) Cα-Backbone root mean square deviation (RMSD) of the human DPP4 protein during 150 ns of the molecular dynamics simulation period. (b) Cα-Backbone root mean square deviation (RMSD) of MERS-CoV S1 RBD protein during 150 ns of the molecular dynamics simulation period. (c) Cα-Backbone root mean square fluctuation (RMSF) of MERS-CoV S1 RBD protein during 150 ns of the molecular dynamics simulation period. (TIF) [file pone.0258750.s002.tif]

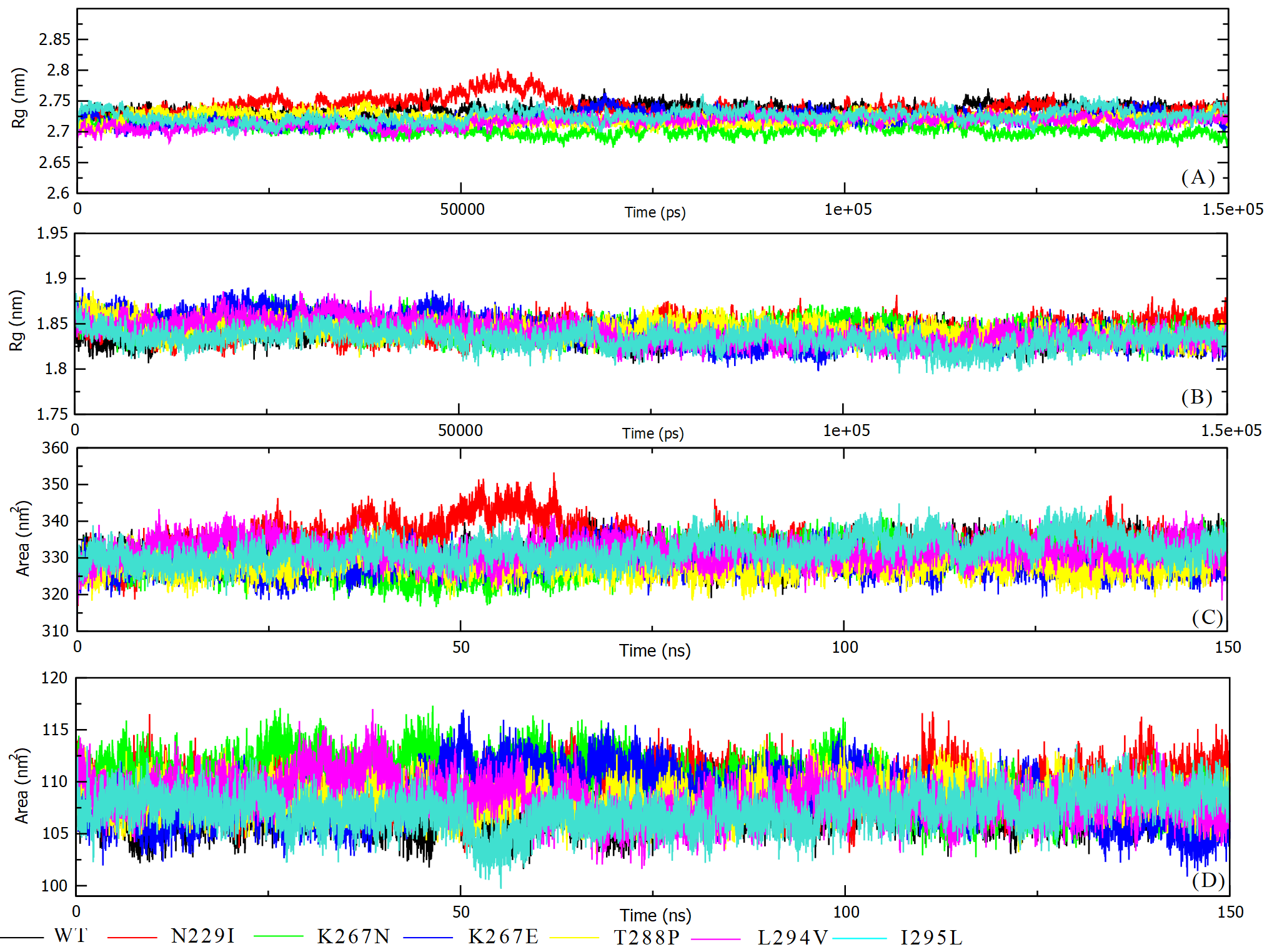

Supplement: S3 Fig — (a) Cα-Backbone Radius of gyration (Rg) of the human DPP4 protein during 150 ns of the molecular dynamics simulation period. (b) Cα-Backbone Radius of gyration (Rg) of MERS-CoV S1 RBD protein during 150 ns of the molecular dynamics simulation period. (c) Solvent accessible surface area (SASA) of the human DPP4 protein during 150 ns of the molecular dynamics simulation period. (d) Solvent accessible surface area (SASA) of the MERS-CoV S1 RBD protein during 150 ns of the molecular dynamics simulation period. (TIF) [file pone.0258750.s003.tif]

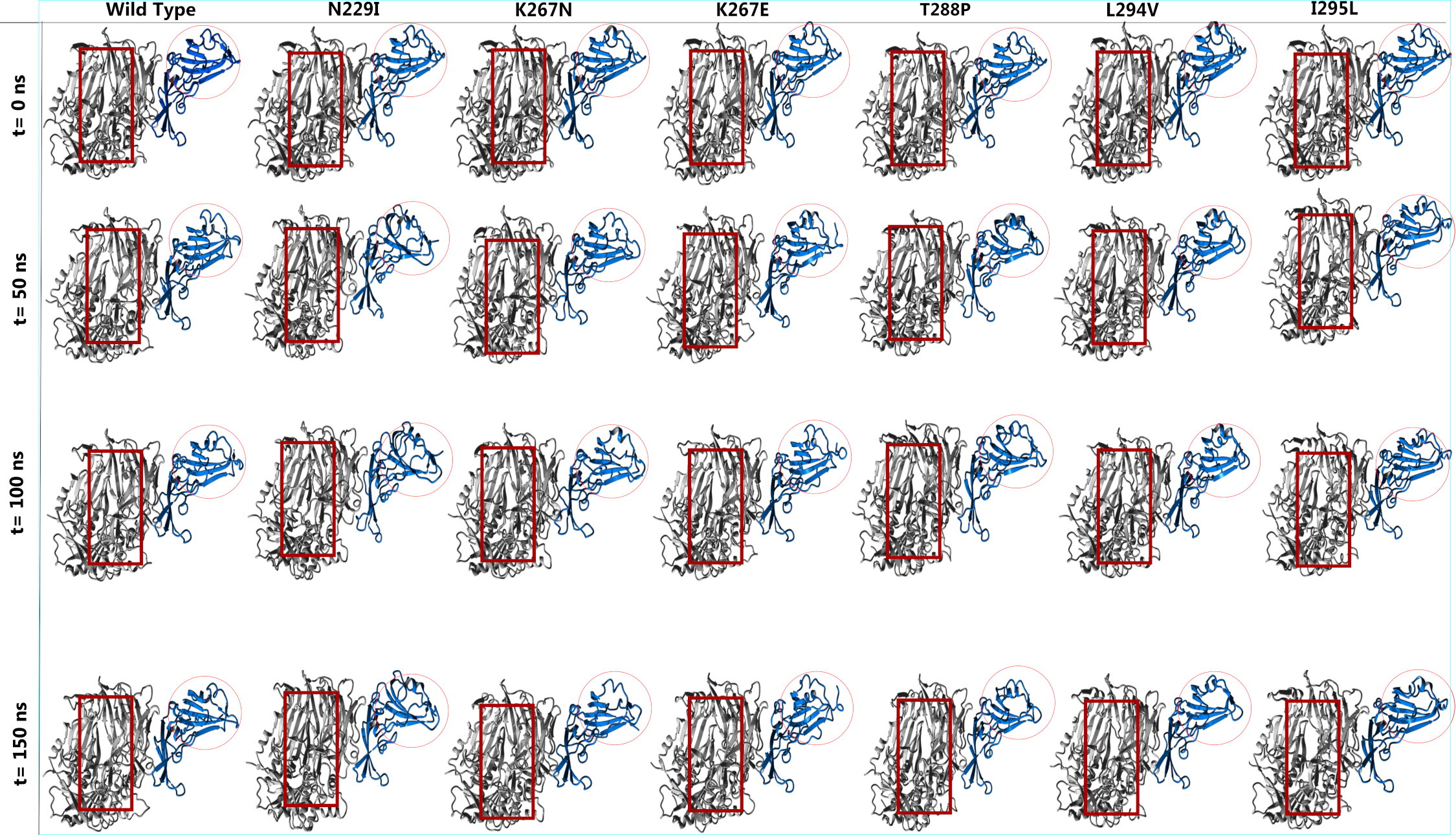

Supplement: S4 Fig — DPP4 and MERS-CoV S protein are dyed respectively with grey and blue. Local structural changes during MD simulation in DPP4 blade IV and V were highlighted in a red square, while local structural changes during MD simulation in MERS-CoV S1 RBD were highlighted in a red circle. (TIF) [file pone.0258750.s004.tif]
