# Supplementary material for: Effect of identified non-synonymous mutations in DPP4 receptor binding residues among highly exposed human population in Morocco to MERS-CoV through computational approach
Source: PLoS One. 2021 Oct 14;16(10):e0258750. doi: 10.1371/journal.pone.0258750 (PMC8516309; doi:10.1371/journal.pone.0258750)
Supplement: S1 Table — (DOCX) [file pone.0258750.s005.docx]

S1 Table: DPP4 structure model validation values using ProSA and Verify 3D web server.

| *DPP4 structure model* | *ProSA z-score* | *Verify3D* |
| --- | --- | --- |
| DPP4 – WT | -10.84 | 94.42% |
| DPP4 – N229I | -10.84 | 94.53% |
| DPP4 – K267N | -10.82 | 89.16% |
| DPP4 – K267E | -10.85 | 90.77% |
| DPP4 – T288P | -10.84 | 94.53% |
| DPP4 – L294V | -10.84 | 94.42% |
| DPP4 – I295L | -10.87 | 94.53% |
