# Supplementary material for: Effect of identified non-synonymous mutations in DPP4 receptor binding residues among highly exposed human population in Morocco to MERS-CoV through computational approach
Source: PLoS One. 2021 Oct 14;16(10):e0258750. doi: 10.1371/journal.pone.0258750 (PMC8516309; doi:10.1371/journal.pone.0258750)
Supplement: S2 Table — (DOCX) [file pone.0258750.s006.docx]

S2 Table: Docking validation values of DPP4 – MERS-CoV S1 RBD complex structure model using HADDOCK 2.4 webserver.

| *DPP4 – MERS-CoV S1 RBD complex structure model* | *HADDOCK Score* | *z-score* | *RMSD from the overall lowest-energy structure* | *Van der Waals energy* | *Electrostatic energy* | *Desolvation energy* | *Restraints violation energy* | *Buried Surface Area* |
| --- | --- | --- | --- | --- | --- | --- | --- | --- |
| 4L72 – WT | -145.8 +/- 2.2 | -1.0 | 0.6 +/- 0.4 | -67.8 +/- 8.0 | -487.7 +/- 29.5 | 5.5 +/- 4.5 | 10.8 +/- 9.3 | 2510.3 +/- 81.0 |
| 4L72 – N229I | -136.7 +/- 4.2 | -1.5 | 0.8 +/- 0.5 | -65.5 +/- 6.5 | -453.8 +/- 44.5 | 5.2 +/- 2.8 | 13.6 +/- 12.2 | 2379.4 +/- 121.7 |
| 4L72 – K267N | -136.7 +/- 1.8 | -1.0 | 0.5 +/- 0.3 | -84.8 +/- 3.4 | -398.5 +/- 24.0 | -1.5 +/- 3.5 | 11.2 +/- 7.4 | 2762.8 +/- 68.4 |
| 4L72 – K267E | -139.4 +/- 1.3 | -1.0 | 0.4 +/- 0.3 | -90.1 +/- 4.2 | -391.0 +/- 22.0 | -2.6 +/- 1.5 | 10.4 +/- 2.5 | 2798.8 +/- 82.4 |
| 4L72 – T288P | -143.8 +/- 3.4 | -1.3 | 0.6 +/- 0.4 | -70.3 +/- 10.0 | -474.3 +/- 46.4 | 2.1 +/- 3.2 | 9.4 +/- 9.7 | 2569.5 +/- 113.8 |
| 4L72 – L294V | -142.7 +/- 2.8 | -1.8 | 0.6 +/- 0.4 | -66.4 +/- 4.8 | -486.6 +/- 30.6 | 4.8 +/- 1.4 | 6.4 +/- 3.5 | 2493.0 +/- 139.9 |
| 4L72 – I295L | -138.3 +/- 2.8 | -2.1 | 0.6 +/- 0.4 | -67.1 +/- 5.2 | -487.8 +/- 27.2 | 7.0 +/- 0.7 | 20.0 +/- 13.7 | 2509.5 +/- 148.8 |
