# Supplementary material for: Effect of identified non-synonymous mutations in DPP4 receptor binding residues among highly exposed human population in Morocco to MERS-CoV through computational approach
Source: PLoS One. 2021 Oct 14;16(10):e0258750. doi: 10.1371/journal.pone.0258750 (PMC8516309; doi:10.1371/journal.pone.0258750)
Supplement: S3 Table — (DOCX) [file pone.0258750.s007.docx]

S3 Table: Summary of DPP4 – MERS-CoV S1 RBD complex structure validation parameters before molecular dynamics simulation production.

| *DPP4 – MERS-CoV S1 RBD structure model* | *Ions neutralisation* | *Energy potential (kJ/mol)* | *Temperature (K)* | *Pressure (bar)* | *Density (kg/m3)* |
| --- | --- | --- | --- | --- | --- |
| 4L72 – WT | 16 Na^+^ | -3.29761e+06 | 299.758 | 1.0 | 1022.93 |
| 4L72 – N229I | 17 Na^+^ | -3.15036e+06 | 299.884 | 1.02 | 1023.94 |
| 4L72 – K267N | 19 Na^+^ | -3.18516e+06 | 299.829 | 1.01 | 1023.74 |
| 4L72 – K267E | 20 Na^+^ | -3.24791e+06 | 299.868 | 1.01 | 1023.24 |
| 4L72 – T288P | 17 Na^+^ | -3.19939e+06 | 299.854 | 1.01 | 1023.29 |
| 4L72 – L294V | 18 Na^+^ | -3.17617e+06 | 299.751 | 1.0 | 1023.8 |
| 4L72 – I295L | 17 Na^+^ | -3.15084e+06 | 299.76 | 1.0 | 1023.6 |
