# Supplementary material for: Effect of identified non-synonymous mutations in DPP4 receptor binding residues among highly exposed human population in Morocco to MERS-CoV through computational approach
Source: PLoS One. 2021 Oct 14;16(10):e0258750. doi: 10.1371/journal.pone.0258750 (PMC8516309; doi:10.1371/journal.pone.0258750)
Supplement: S4 Table — (DOCX) [file pone.0258750.s008.docx]

**S4 Table: Molecular dynamics analysis parameters (RMSD, RMSF, Rg, SASA and H-bond) mean values and standard deviation for each complex model and individual component, i.e: DPP4 and MERS-CoV S1 RBD protein.**

| *Model* | *RMSD (nm)* | | *RMSF (residue)* | | *Rg (nm)* | | *SASA (nm²)* | | *H-bond (number)* | |
| --- | --- | --- | --- | --- | --- | --- | --- | --- | --- | --- |
|  | *Mean value* | *Standard Deviation* | *Mean value* | *Standard Deviation* | *Mean value* | *Standard Deviation* | *Mean value* | *Standard Deviation* | *Mean value* | *Standard Deviation* |
| *4L72-WT* | 0.2519 | 0.0628 | 1.025 | 0.137 | 3.308 | 0.019 | 419.274 | 4.331 | 675.99 | 12.349 |
| *4L72-N229I* | 0.5322 | 0.089 | 1.113 | 0.154 | 3.35 | 0.018 | 421.867 | 5.492 | 653.223 | 14.064 |
| *4L72-K267N* | 0.228 | 0.033 | 0.48 | 0.128 | 3.288 | 0.021 | 419.009 | 4.331 | 672.022 | 13.689 |
| *4L72-K267E* | 0.254 | 0.042 | 0.751 | 0.14 | 3.292 | 0.033 | 418.077 | 5.662 | 672.078 | 12.361 |
| *4L72-T288P* | 0.295 | 0.059 | 0.627 | 0.109 | 3.333 | 0.012 | 416.125 | 3.515 | 666.154 | 14.139 |
| *4L72-L294V* | 0.271 | 0.035 | 0.505 | 0.087 | 3.318 | 0.018 | 418.759 | 4.874 | 682.509 | 14.785 |
| *4L72-I295L* | 0.262 | 0.025 | 0.452 | 0.092 | 3.304 | 0.013 | 417.458 | 5.175 | 671.677 | 12.907 |
| *DPP4-WT* | 0.2707 | 0.059 | 0.91 | 0.091 | 2.735 | 0.008 | 331.461 | 3.103 | NA | NA |
| *DPP4-N229I* | 0.473 | 0.080 | 0.99 | 0.092 | 2.739 | 0.014 | 334.387 | 4.766 | NA | NA |
| *DPP4-K267N* | 0.2508 | 0.0244 | 0.34 | 0.050 | 2.704 | 0.0103 | 329.33 | 3.863 | NA | NA |
| *DPP4-K267E* | 0.2580 | 0.033 | 0.61 | 0.062 | 2.721 | 0.010 | 328.66 | 2.947 | NA | NA |
| *DPP4-T288P* | 0.286 | 0.045 | 0.5 | 0.056 | 2.729 | 0.008 | 328.017 | 2.858 | NA | NA |
| *DPP4-L294V* | 0.2708 | 0.026 | 0.4 | 0.046 | 2.716 | 0.008 | 331.784 | 3.084 | NA | NA |
| *DPP4-I295L* | 0.2786 | 0.0276 | 0.35 | 0.047 | 2.725 | 0.010 | 332.153 | 3.618 | NA | NA |
| *MERS-CoV S1 RBD-WT* | 0.299 | 0.09 | 0.115 | 0.046 | 1.837 | 0.009 | 107.143 | 1.59 | NA | NA |
| *MERS-CoV S1 RBD -N229I* | 0.841 | 0.14 | 0.123 | 0.062 | 1.844 | 0.01 | 109.66 | 1.773 | NA | NA |
| *MERS-CoV S1 RBD -K267N* | 0.352 | 0.062 | 0.14 | 0.078 | 1.844 | 0.01 | 110.07 | 2.233 | NA | NA |
| *MERS-CoV S1 RBD -K267E* | 0.448 | 0.062 | 0.141 | 0.078 | 1.842 | 0.015 | 108.518 | 2.367 | NA | NA |
| *MERS-CoV S1 RBD -T288P* | 0.499 | 0.088 | 0.127 | 0.053 | 1.842 | 0.009 | 108.456 | 1.491 | NA | NA |
| *MERS-CoV S1 RBD -L294V* | 0.414 | 0.065 | 0.105 | 0.041 | 1.84 | 0.012 | 108.372 | 1.913 | NA | NA |
| *MERS-CoV S1 RBD -I295L* | 0.403 | 0.058 | 0.102 | 0.045 | 1.833 | 0.01 | 107.24 | 1.749 | NA | NA |
| *WT-DPP4- MERS-CoV S1 RBD (interface)* | NA | NA | NA | NA | NA | NA | NA | NA | 8.012 | 2.672 |
| *N229I-DPP4- MERS-CoV S1 RBD (interface)* | NA | NA | NA | NA | NA | NA | NA | NA | 4.178 | 2.622 |
| *K267N-DPP4- MERS-CoV S1 RBD (interface)* | NA | NA | NA | NA | NA | NA | NA | NA | 9.202 | 2.683 |
| *K267E-DPP4- MERS-CoV S1 RBD (interface)* | NA | NA | NA | NA | NA | NA | NA | NA | 6.816 | 2.225 |
| *T288P-DPP4- MERS-CoV S1 RBD (interface)* | NA | NA | NA | NA | NA | NA | NA | NA | 5.429 | 2.062 |
| *L294V-DPP4- MERS-CoV S1 RBD (interface)* | NA | NA | NA | NA | NA | NA | NA | NA | 6.994 | 2.284 |
| *I295L-DPP4- MERS-CoV S1 RBD (interface)* | NA | NA | NA | NA | NA | NA | NA | NA | 6.815 | 2.843 |

- NA : Not Applicable
